# Synaptic Vesicle Glycoprotein 2A Suppresses Amyloidogenesis Beyond Its Synaptic Role: A Novel Mechanism Disrupting BACE1 Binding and Altering APP Localization

**DOI:** 10.1111/acel.70379

**Published:** 2026-01-11

**Authors:** Xiaoling Wang, Qian Zhang, Xiaomin Zhang, Jing Liu, Jingjing Zhang, Congcong Liu, Yuting Cui, Qiao Song, Yuli Hou, Yaqi Wang, Min Cao, Peichang Wang

**Affiliations:** ^1^ Department of Clinical Laboratory, Xuanwu Hospital, National Clinical Research Center for Geriatric Disorders Capital Medical University Beijing China; ^2^ Department of Clinical Laboratory Changzhi People's Hospital of Changzhi Medical College Changzhi Shanxi China; ^3^ Department of Clinical Laboratory Beijing Huairou Hospital Beijing China

**Keywords:** β‐Site amyloid precursor protein cleaving enzyme 1, alzheimer's disease, amyloid degradation, amyloid precursor protein, synaptic vesicle glycoprotein 2A

## Abstract

Synaptic vesicle glycoprotein 2A (SV2A), a transmembrane protein widely localized to synaptic vesicles, serves as a key indicator of synaptic loss in Alzheimer's disease (AD). In this study, adeno‐associated virus (AAV) was injected by brain stereotactic injection technique to construct SV2A‐overexpressing APP/PS1 mice, then the effects of SV2A on amyloid precursor protein (APP) degradation and its molecular mechanism were further explored in vivo or in vitro. Our results demonstrated that SV2A overexpression significantly reduced Aβ plaque deposition in brain tissue of APP/PS1 mice. Mechanistically, SV2A was identified as a novel APP‐binding protein that attenuated the amyloidogenic processing of APP by inhibiting its interaction with β‐site APP cleaving enzyme 1 (BACE1). Furthermore, SV2A overexpression altered the subcellular distribution of APP, shifting its localization away from the endosomal‐lysosomal compartments. Collectively, our findings unveil SV2A as a critical regulator of APP metabolism and propose it as a promising therapeutic target for intervening in the early pathological progression of AD.

## Graphical abstract

Synaptic vesicle glycoprotein 2A, the newly identified APP‐binding protein, reduces amyloid‐β plaque deposition in Alzheimer's disease by suppressing the amyloidogenic pathway through inhibition of BACE1‐APP interaction and alteration of APP endosomal‐lysosomal localization.

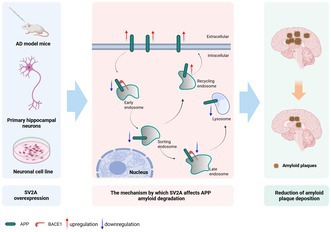

## 1 Introduction

Currently, an estimated 47 million people worldwide suffer from Alzheimer's disease (AD) (Dubois et al. 2021), with official statistics of 119,399 deaths due to AD in 2021, and the cost of healthcare and care for people with dementia has already reached $346.6 billion in 2023 alone, which places a heavy burden on society and families (2024 Alzheimer's disease facts and figures 2024). AD is a typical progressive neurodegenerative disease characterized by cognitive impairment with distinctive neuropathology that includes the deposition of senile plaques, neurofibrillary tangles, synapses, and neuron loss (2023 Alzheimer's disease facts and figures 2023; Mohanty et al. 2022; Wang et al. 2024; Zhu et al. 2023). Studies have demonstrated that senile plaques are mainly composed of the β‐amyloid (Aβ) peptide, which is generated through the sequential cleavage of the APP by BACE1 and γ‐secretase (Choi et al. 2023; Musardo et al. 2022; Nakamura et al. 2021; Serneels et al. 2020; Vilella et al. 2024; Zhou et al. 2023). β‐amyloid plaques in the brain seem to be a key initiating event in AD (Ng et al. 2024; Senapati et al. 2024), which possess strong toxicity that can activate microglia, induce neuroinflammatory response, cause neurotoxic effects, and subsequently lead to a series of AD pathological manifestations (Calvo‐Rodriguez et al. 2024; Kuhn et al. 2024; Kumar et al. 2024; Senapati et al. 2024). In addition, it has been suggested that the onset of amyloid deposition may occur ~20 years prior to cognitive alterations (Jansen et al. 2015), and the gradual progression of Aβ deposition in the brain subsequently triggers the spread of tau pathology, ultimately leading to neuronal loss (Karran and De Strooper 2022). Recently, the role of Aβ protein aggregation in the pathogenesis of AD has been further highlighted with the successive advances in clinical trials of anti‐amyloid immunotherapies such as aducanumab (Dhillon 2021) and lecanemab (van Dyck et al. 2023), which triggered us to seek early events in AD and early treatment targets so as to try to “stop Alzheimer's disease before it starts” (McDade and Bateman 2017).

APP, the precursor substance of Aβ, is a type I transmembrane protein that is highly expressed in neuronal dendrites and axons (Montenegro et al. 2023). The way in which APP is degraded, mainly categorized into the amyloid metabolic pathway and non‐amyloid degradation pathway, directly affects the progression of AD (Haass et al. 2012). The binding protein of APP is the key factor influencing its degradation, which provides us with new ideas to find the triggering mechanism of Aβ deposition and may become potential targets for early AD treatment.

SV2A, a 12‐transmembrane protein mainly present on synaptic vesicles, is an important member of the synaptic vesicle glycoprotein 2 (SV2) family (Bajjalieh et al. 1994; Liu et al. 2023). SV2A is widely expressed in excitatory and inhibitory neurons in the hippocampus, cortex, and cerebellum and plays an important role in the circulation of synaptic vesicles and in regulating the release of action potential‐dependent neurotransmitters (Janz et al. 1999). Recently, several studies have found that SV2A positron emission tomography (PET) imaging could be used as a biomarker of synaptic degeneration and AD pathology (Onwordi et al. 2024; Whiteside et al. 2023), which revealed the role of SV2A in AD progression. Although O'Dell et al. (2021) observed a significant inverse association between global Aβ deposition and hippocampal SV2A binding in participants with aMCI by PET, the detailed molecular mechanisms between SV2A and APP as well as Aβ were not elucidated.

In this study, SV2A was initially observed to reduce Aβ protein levels and amyloid deposition in neuronal cells and AD model mice. Further investigation into the underlying mechanisms revealed that SV2A, as a binding protein of APP, could reduce amyloidosis of APP by inhibiting the binding of BACE1 to APP and regulating the intracellular distribution of APP. Considering the biological role of SV2A and its progressive decrease with AD progression, it has the potential to be a key target for early intervention of AD.

## 2 Material and Methods

### 2.1 Subjects

In total, the CSF specimens were collected from 112 subjects, which included 45 controls, 13 aMCI, 41 AD, and 13 VaD. In addition, the serum specimens were collected from 358 subjects, which included 95 healthy controls, 80 aMCI, 145 AD, and 38 VaD. The diagnoses of AD were based on the criteria published by the National Institute on Aging and Alzheimer's Association (NIA‐AA) (McKhann et al. 2011). aMCI was diagnosed by the criteria described previously (Gauthier et al. 2006). The diagnosis of VaD was based on previously published criteria (Roman et al. 1993). All diagnoses were made by at least two experienced physicians of the Department of Neurology, Xuanwu Hospital, Capital Medical University.

The control subjects undergoing CSF testing were first‐time hospitalized patients who had not undergone systemic therapy, did not have any major systemic illnesses such as tumors or diabetes, and had no cognitive impairments based on the diagnosis of at least two experienced neurologists in conjunction with imaging tests. Control subjects for serum testing were age‐matched subjects from the Physical Examination Center of Xuanwu Hospital Capital Medical University who were not suffering from tumors, diabetes mellitus, or any neurological disorders. There was no statistically significant difference in terms of age and sex between the dementia and control groups. Clinical and demographic features of the diagnostic cohorts are shown in Table S2. This study was approved by the ethics committee of Xuanwu Hospital Capital Medical University, Beijing, China (Lin‐Yan‐Shen [2021] No. 225), and was conducted in accordance with the principles of the Declaration of Helsinki. Written informed consent was obtained from all participants or their legal guardians.

### 2.2 CSF and Serum Collection

CSF samples were collected according to the international guidelines. Briefly, the participants were placed in the left lateral position for lumbar puncture, and the L3–L5 intervertebral disc spaces were selected as the puncture site. The CSF samples were collected using a 20‐gauge atraumatic needle and centrifuged at 2000 × *g* for 10 min at room temperature.

Blood samples were drawn via venipuncture in the morning time after a 12‐h fast and were stored in serum tubes containing a clot activator. Sera were extracted from the blood samples by centrifugation at 2000 × *g* for 10 min. All samples were stored in polypropylene tubes at −80°C until use to avoid repeated freezing and thawing.

### 2.3 Animal Models

AD model transgenic mice overexpressing mutant familial AD genes (APP/presenilin‐1‐dE9, APP/PS1) were purchased from Zhishan Healthcare Research Institute Ltd. (Beijing, China). All animal experiments conformed to the National Institutes of Health guidelines and were approved by the Ethics Committee of Xuanwu Hospital Capital Medical University (Lin‐Yan‐Shen [2021] No. 225).

### 2.4 Simoa Assay for CSF and Serum SV2A Test

For SV2A Simoa assay, the polyclonal antibody specifically recognizing SV2A was used (CSB‐PA022978LA01HU, CUSABIO, Wuhan, China) as the capture antibody, with identifying sequence of 36–149, and another rabbit anti‐SV2A antibody (bs‐2407R, BIOSS, Beijing, China) used as the detection antibody with the recognition site of 451–550. The CSF and serum samples were analyzed on the AST‐Sc‐Lite, a fully‐auto single‐molecule detection machine (Suzhou AstraBio Technology Co. Ltd., China), according to the manufacturer's instructions. The testing process is described elsewhere (Tao et al. 2024).

### 2.5 Lentivirus and Plasmids

The lentivirus and plasmids encoding SV2A full‐length cDNA (SV2Aoe) (human, GenBank ID: NM_001328674.2) and the control were purchased from Hanbio Biotechnology (Shanghai, China). The lentivirus and plasmids encoding SV2A full‐length cDNA (SV2Aoe) (mouse, GenBank ID: NM_022030) and the control were purchased from SyngenTech (Beijing, China).

The plasmids encoding full‐length App cDNA (NM_001198823) were cloned into the NotI/XbaI sites of the pcDNA4/myc‐His B vector. The plasmids encoding full‐length Med23 cDNA (NM_001166416) were cloned into the NotI/XbaI sites of the p3xFLAG‐CMV‐10 vector. The plasmids encoding full‐length Clu cDNA (NM_013492), Epn1 cDNA (NM_001252454), Homer1 cDNA (NM_031707), Nefh cDNA (NM_010904.3), and Sv2a cDNA (NM_022030) were cloned into the HindIII/EcoRI sites of the p3xFLAG‐CMV‐10 vector. The plasmids encoding full‐length Adamts7 cDNA (NM_001003911), Hnrnph2 cDNA (NM_001313716), Sh3gl2 cDNA (NM_019535), Synj1 cDNA (NM_001045515), and Vps35 cDNA (NM_022997) were cloned into the NotI/KpnI sites of the p3xFLAG‐CMV‐10 vector.

### 2.6 Cell Culture and Transfection

The SH‐SY5Y cells were maintained in a MEM medium supplemented with 10% FBS and 1% penicillin–streptomycin at 37°C in a humidified incubator containing 5% CO_2_. The N2a cells were maintained in DMEM supplemented with 10% FBS and 1% penicillin/streptomycin at 37°C in a humidified incubator containing 5% CO_2_. The plasmids were transfected with jetOPTIMUS reagent (Polyplus‐transfection Inc., USA) at a confluency of 60%–80%. The HEK293T cells were maintained in DMEM supplemented with 10% FBS and 1% penicillin/streptomycin at 37°C in a humidified incubator containing 5% CO_2_. The plasmids were transfected with jetOPTIMUS reagent (Polyplus‐transfection Inc., USA) at a confluency of 60%–80%. The plasmids were synthesized by Sangon Biotech (Shanghai, China) Company.

Primary neurons were isolated from the cortex and hippocampus of APP/PS1 mice on embryonic day 17 (E17). After separation and digestion, the cells were cultured on plates in a neurobasal medium (Invitrogen) containing a 2% B27 supplement (Invitrogen), 0.25% glutamine, and 0.5% penicillin/streptomycin (Invitrogen) and incubated at 37°C in a 5% CO_2_ incubator. Subsequent experiments were conducted after 7 days of culture. The primary hippocampal neurons were infected by lentivirus.

### 2.7 Co‐Immunoprecipitation Assay

The cells and mouse brain tissue were lysed in an ice‐cold lysis buffer containing 0.01 M PBS, pH 7.4, 2 mM EDTA, and 1% Triton X‐100 supplemented with protease and phosphatase inhibitor cocktails (Invitrogen). Antibodies or mouse IgG isotype control (Invitrogen, USA) were added to the precipitation system and rotated at 4°C overnight. Protein A/G beads (Millipore, Billerica, MA, USA) were sequentially added and incubated for 30 min at room temperature with continuous mixing to capture the immune complex. The beads were washed several times, and the immunoprecipitated proteins were resolved by 1× sodium dodecyl sulfate‐polyacrylamide gel electrophoresis (SDS–PAGE) and analyzed by immunoblotting.

### 2.8 Protein Extraction and Western Blotting

The brain tissues or cells were lysed in 0.01 M PBS (pH 7.4), 2 mM EDTA, 1% Triton X‐100 lysis buffer (Solarbio, Shanghai, China) supplemented with protease and phosphatase inhibitor cocktails (Thermo Fisher Scientific, USA). The protein concentration was determined using a BCA Protein Assay kit (Thermo Fisher Scientific). Equal amounts of protein were loaded onto 10% (or 12%) SDS–PAGE gels and separated by electrophoresis. The isolated proteins were transferred onto PVDF membranes (Millipore). The membranes were blocked with 5% w/v nonfat milk for 1 h at room temperature. The membranes were incubated with primary antibodies in TBST containing 5% w/v nonfat milk overnight at 4°C. The next day, the membranes were washed thrice for 5 min each time with TBST at room temperature. Then, the membranes were incubated with the corresponding horseradish peroxidase‐conjugated secondary antibodies (ZSGB‐BIO, Beijing, China) in TBST containing 5% w/v nonfat milk for 1 h at room temperature. After washing extensively with TBST, the protein bands were visualized using a Super ECL Detection Reagent (Yeasen, Shanghai, China) and imaged using the Tanon 4600SF. The bands were quantified with Image J software, and the level of the target protein was normalized to that of β‐actin or GAPDH. The following antibodies were used for this experiment: anti‐SV2A (Cat. No. ab32942, Abcam, Cambridge, MA, USA, 1:3000); anti‐APP (Cat. No. ab32136, Abcam, 1:5000); IgG isotype control (Cat. No. 10400C, Invitrogen, Carlsbad, CA, USA, 1:5000); anti‐β‐actin (Cat. No. TA‐09, ZSGB‐BIO, Shanghai, China, 1:8000), anti‐GAPDH (Cat. No. 60004‐1‐Ig, Proteintech, Wuhan, China, 1:8000), anti‐BACE1 (Cat. No. ab183612, Abcam, 1:800); anti‐EEA1 (Cat. No. 610456, BD Biosciences, 1:1000); anti‐Rab7 (ab137029, Abcam, 1:2000); anti‐Rab11 (Cat. No. 5589, Cell Signaling Technology, 1:1000); anti‐LAMP1 (Cat. No. sc2011, Santa Cruz, 1:1000); myc tag polyclonal antibody (Cat. No. 16286‐1‐AP, Proteintech, Wuhan, China, 1:3000); DYKDDDDK tag polyclonal antibody (20543‐1‐AP, Proteintech, Wuhan, China, 1:3000).

### 2.9 Thioflavin S Staining

The deposition area and the number of amyloid plaques in the mouse brain were observed using Thioflavin S staining. Briefly, the brain sections were incubated in filtered 0.3% Thioflavin S solution for 8 min at room temperature. The sections were washed in 50% ethanol and 1 × TBS buffer. Then, the brain sections were observed under a Nikon light microscope after sealing the tissues on the slides with neutral balsam.

### 2.10 Elisa

The levels of Aβ40 (Cat. No. EM0863, FineTest, Wuhan, China), Aβ42 (Cat. No. EM0864, FineTest, Wuhan, China), sAPPα (Cat. No. ml625251V, Mlbio, Shanghai, China), and sAPPβ (Cat. No. ml057880V, Mlbio, Shanghai, China) were measured by ELISA in the brain tissue lysates and serum from APP/PS1 mice injected with AAV9‐SV2Aoe or AAV9‐Con and in the supernatants from primary hippocampal neuronal cells infected with lentivirus following the manufacturer's instructions. In addition, the levels of Aβ40 (Cat. No. EH2684, FineTest), Aβ42 (Cat. No. EH2685, FineTest), sAPPα (Cat. No. ml025433, Mlbio), and sAPPβ (Cat. No. ml503721, Mlbio) were measured in the supernatant of SH‐SY5Y‐APP cells transfected with plasmids following the manufacturer's instructions.

### 2.11 Bimolecular Fluorescence Complementation Assay (BiFC)

SH‐SY5Y cells were transfected using Xfect Transfection Reagent approximately 24 h after plating in accordance with the manufacturer's instructions. The BiFC experiment was performed with reference to a previously published study (Zhuravleva et al. 2021). Briefly, double transfection of BACE1:VC construct (pZDonor‐CMV‐BACE1‐linker‐VenusC155‐BGH_pA‐hef1a‐mKate‐terminator) and APP: VN construct (pZDonor‐CMV‐APP‐linker‐VenusN155‐BGH_pA‐hef1a‐EBFP2‐terminator constructs) was performed 24 h after transfection with SV2Aoe plasmid. The cells were then fixed and analyzed 24 h after the APP: VN and BACE1:VC transfection.

### 2.12 Cell Surface Biotinylation Assay

Surface biotinylation assay was performed on SH‐SY5Y according to the instruction (Pierce Cell Surface Protein Biotinylation and Isolation Kit, Thermo Fisher Scientific). Briefly, SH‐SY5Y cells transfected with plasmids were first incubated with Sulfo‐NHS‐SS‐Biotin at 4°C for 30 min. Then, the cells were washed twice with TBS to remove the excess biotin. Following the washing steps, the cells were harvested in a lysis buffer, with protein extraction and concentration measurement performed as mentioned earlier. A portion of the supernatant was collected and used as the input. To precipitate the biotinylated proteins, 200 μg of the supernatant was incubated with NeutrAvidin Agarose for 30 min at room temperature. Following the incubation, the beads were washed and incubated with elution buffer mixed with the DTT stock solution for 30 min at room temperature to elute proteins. The eluate was added to SDS–PAGE and heated at 70°C for 10 min, followed by analyses by Western blotting using the corresponding antibodies (APP: Cat. No. ab32136, Abcam, USA).

### 2.13 Stereotaxic Injection of Adeno‐Associated Virus (AAV)

For the construction of AAV9‐SV2Aoe, the vector HBAAV9‐m‐3 × flag‐T2A‐mcherry (Hanbio Biotechnology, Shanghai, China) was used. Briefly, 9‐month‐old APP/PS1 mice prepared for the stereotaxic injection were deeply anesthetized with an intraperitoneal injection of sodium pentobarbital and secured in a stereotaxic apparatus. The AAV9 was bilaterally injected into the dorsal hippocampal CA1 area at a rate of 0.2 μL/min, and the total volumes of AAV‐SV2Aoe (1.3 × 10^12^ vg/mL) and AAV‐Con (1.9 × 10^12^ vg/mL) injected into the unilateral hippocampal region were 1.5 μL and 1.0 μL, respectively.

### 2.14 Novel Object Recognition Test (NOR)

NOR experiments were performed 5 weeks after the AAV injection. The mice were monitored by a digital camera placed above the test chamber and connected to a video tracking system (Smart v3.0). The mice were taken into the behavioral room 1 h before the training and test sessions to avoid the stress. In the first 2 days of NOR, the training session was performed by exposing the mice for 10 min to two copies of the same object that cannot be moved. Prior to the training, the experimenter ensured that the chamber was clean and odorless and the mice were placed individually at the mid‐point of the wall opposite to the sample objects with the nose pointing away from the objects. At the end of the training, the experimental mice were placed in other pre‐prepared rearing cages. The apparatus unit was deodorized by spraying it with alcohol and drying it with a paper towel. After 24 h of the training session, the test session was performed by exposing the mice for 10 min to one of the objects used in the training session and a second novel object. The time of retention and the number of entries in the area around the new and old objects during the test period were recorded separately, and the cognitive index of the mice was subsequently calculated.

### 2.15 Morris Water Maze Test (MWM)

MWM test was conducted as described previously. Briefly, the mice were trained in the water maze for 5 consecutive days. The time required to reach the platform (escape latency) was measured. If the mice were unable to reach the platform within 60 s, they were gently guided to the platform and left there for 20 s. When the 5‐day training was completed, the platform was removed from the pool, and the mice were subjected to a probe trial session on the 6th day. The mice were then allowed to swim for 60 s to search for the platform, and their motion trajectories were recorded with a video camera and then analyzed by a computer video system.

### 2.16 Histology and Immunostaining

Mice brain tissues fixed with 4% PFA were dehydrated in 30% sucrose and embedded in the optimal cutting temperature compound for frozen sectioning. Antigen retrieval was performed by using citric acid (pH 6.0) antigen repair solution at a sub‐boiling temperature. Then, the sections were immersed in 3% hydrogen peroxide to block the endogenous peroxidase activity, followed by incubation in 3% bovine serum albumin solution to block non‐specific binding. The slides were then incubated overnight at 4°C in a wet box with respective primary antibodies diluted in PBS. The primary antibodies used in the study included rabbit anti‐SV2A antibody (Cat. No. ab254351, Abcam, 1:5000), rabbit anti‐APP (Cat. No. ab32136, Abcam, 1:2000; Cat. No. MAB348, Sigma‐Aldrich, St. Louis, MO, USA, 1:2000), rabbit anti‐BACE1 (Cat. No. ab183612, Abcam, 1:1000), and rabbit anti‐6E10 (Cat. No. 803015, BioLegend, USA, 1:1000). For the chemical visualization of primary antibody signal, HRP‐labeled goat anti‐rabbit IgG secondary antibodies (Cat. No. GB23303, Servicebio, China, 1:500) were applied for 50 min at room temperature in the dark, and each antigen was individually visualized using tyramide signal amplification kits (Cat. No. G1226, Servicebio) according to the manufacturer's protocol. Both the first and second antibodies were removed using an EDTA antigen repair resolution before the next antigen was visualized. The sections were then imaged using a slide scanner (PANNORAMIC, 3D HISTECH, Hungary). Co‐localization was quantified by Image J software and presented as Pearson's correlation coefficient and Mander's correlation coefficient.

### 2.17 Cell Immunocytochemistry

Primary neurons or HT22 cells were cultured on cover glasses in 24‐well cell culture plates and fixed in 4% paraformaldehyde (Cat. No. P1110, Solarbio) for 15 min. The cultures were washed with the PBS solution, permeabilized with 0.1% Triton X‐100, and non‐specific binding was blocked with 10% donkey serum for 1 h at room temperature. The cells on the cover glasses were incubated with mouse anti‐APP (Cat. No. MAB348, Sigma‐Aldrich, 1:400) and rabbit anti‐SV2A (Cat. No. ab32942, Abcam) antibodies at 4°C overnight. Following washing, the cells were incubated with Alexa Fluor 488 donkey anti‐rabbit IgG (Cat. No. ab150077, Abcam, 1:1000) or Alexa Fluor 594 donkey anti‐mouse IgG (Cat. No. ab150116, Abcam, 1:1000) for 1 h at room temperature. The cells on the cover glasses were washed in PBS and incubated with DAPI (Cat. No. D9542, Merck, 1:1000) for 10 min.

SH‐SY5Y and N2a cells plated on a coverslip were fixed with 4% paraformaldehyde for 15 min and permeabilized with Triton X‐100 for 10 min (this step is not performed when observing the fluorescence level of APP on the SH‐SY5Y cell surface), and then blocked with 10% normal donkey serum in PBS at room temperature. SH‐SY5Y and N2a cells were incubated with appropriate primary antibodies overnight at 4°C. The following primary antibodies were used in the study, early endosome antigen 1 (EEA1) (Cat. No. sc‐137,130, Santa Cruz, 1:100); APP (Cat. No. ab32136, Abcam, 1:2000; Cat. No. MAB348, Sigma‐Aldrich, 1:400); BACE1 (Cat. No. PA1‐757, Thermo Fisher Scientific, 1:200); Ras‐related protein Rab‐7 (Rab7) (ab137029, Abcam, 1:100); Ras‐related protein Rab‐11 (Rab11) (Cat. No. 5589, Cell Signaling Technology, 1:100); Lysosome‐associated membrane protein‐1 (LAMP1) (Cat. No. 99437, Cell Signaling Technology, 1:100). The next day, the cells were incubated with appropriate secondary fluorescently conjugated antibodies in PBS for 1 h at room temperature in the dark and then incubated with DAPI (Cat. No. D9542, Merck, 1:1000) for 10 min at room temperature. The coverslips were mounted on glass slides with an antifade mounting medium (Cat. No. P0126, Beyotime, China).

Images were acquired using a Zeiss LSM 800 confocal microscope or Leica TCS SP8 confocal microscope. Co‐localization was quantified by Image J software and presented as Pearson's correlation coefficient and Mander's correlation coefficient. For all imaging experiments, the data were analyzed in a blinded manner.

### 2.18 GST Pulldown Assay and Mass Spectrometry

GST pulldown assay was carried out from mouse hippocampus lysates with GST‐labeled APP and control GST protein. Pulldown components were separated by SDS–PAGE and silver staining. Differential bands in SDS PAGE gels were tryptic digested for mass spectrometry with nano LC‐ESILTQ MS/MS (Thermo Fisher Scientific, USA).

### 2.19 Transcripts Per Million (TPM) Analysis of SV2A Expression in Database

SV2A expression data (in TPM) for the entorhinal cortex, hippocampus, and temporal cortex were queried from the AlzData database (http://www.alzdata.org/) from donors with AD and cognitively normal controls. An unpaired *t*‐test with Welch's correction was used to compare the expression levels between the AD and control groups.

### 2.20 Statistical Analysis

The normality of the distribution of the variables was assessed using the Shapiro–Wilk test. Continuous variables were compared between two independent samples using the *t*‐test or the Mann–Whitney *U*‐test. One‐way ANOVA was applied to compare the statistical differences between multiple groups. Categorical data were analyzed using the Chi‐square test. Logistic regression models were employed to compare the continuous variables between different groups after adjusting for age and sex. Spearman's rank‐correlation analyses were performed to assess the correlations between biomarkers with age, sex, or cognitive scale scores. All tests were two‐tailed, and *p* < 0.05 was considered to indicate statistical significance. All analyses were performed using IBM SPSS Statistics version 24 (IBM Corp., Armonk, NY, USA). Data were visualized using Prism 9 (GraphPad, San Diego, CA, USA).

## 3 Results

### 3.1 SV2A Reduced the Burden of Amyloid Plaques in the Brain Regions of APP/PS1 Mice

**FIGURE 1:**
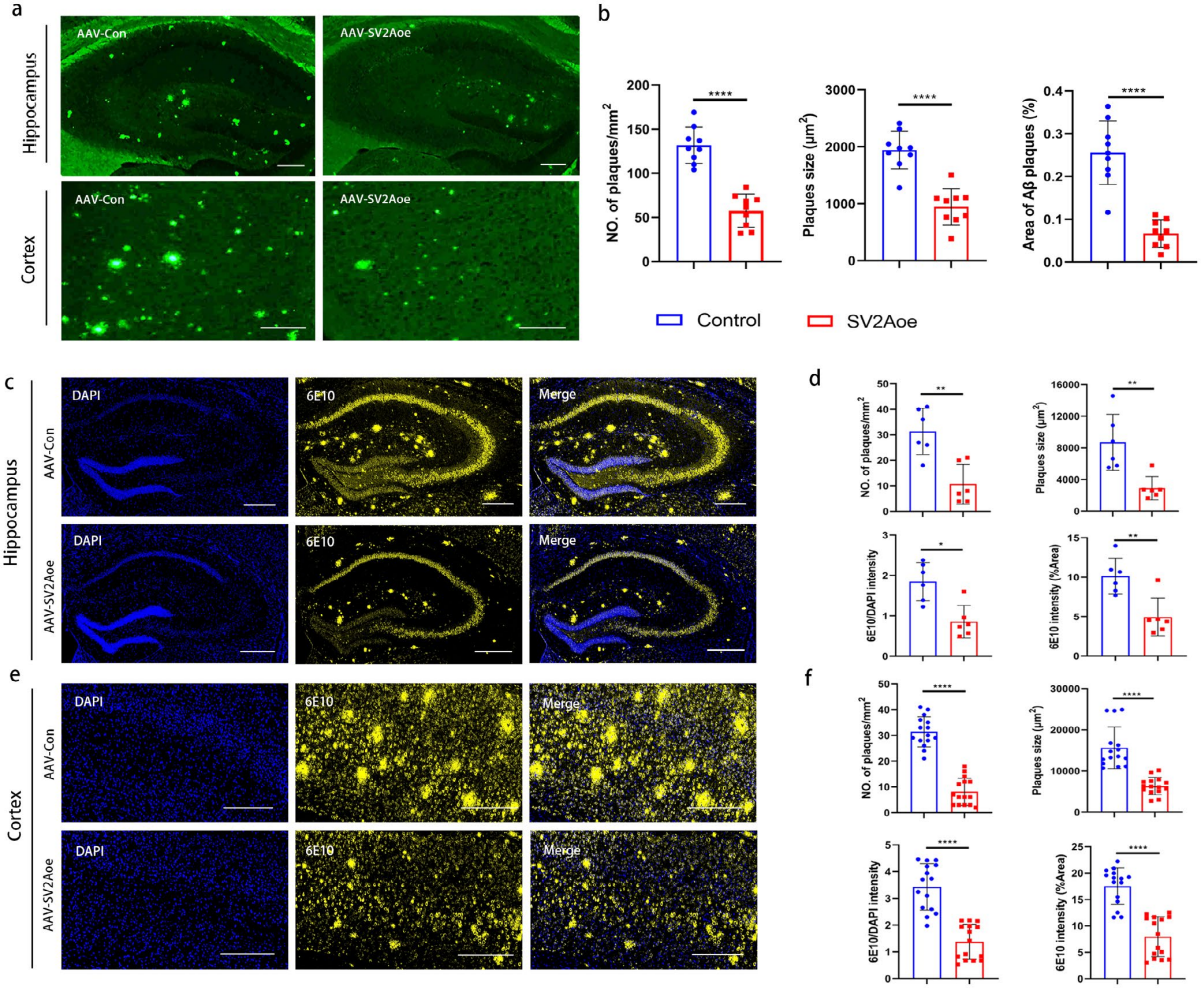
Amyloid deposition in SV2A‐upregulated APP/PS1 mice. (a) Thioflavin S staining of the Aβ plaques in the hippocampus and cortex of APP/PS1 mice injected with AAV‐SV2Aoe or AAV‐Con, Scale bar: 50 μm. (b) Quantification of amyloid plaques per mm^2^ area, amyloid plaque size (μm^2^), and the percentage of Aβ plaques area in brain sections, (*n* = 9 brain sections, from three mice per group). (c) The fluorescence intensity of 6E10 in hippocampus of the APP/PS1 mice injected with AAV‐SV2Aoe or AAV‐Con, Scale bar: 50 μm. (d) Quantification of amyloid plaques per mm^2^ area, amyloid plaque size (μm^2^), the ratio of fluorescence intensity of 6E10 to DAPI and the percentage of 6E10 fluorescence intensity in hippocampal region, (*n* = 6, from three mice per group). (e) The fluorescence intensity of 6E10 in cortex of the APP/PS1 mice injected with AAV‐SV2Aoe or AAV‐Con, Scale bar: 50 μm. (f) Quantification of amyloid plaques per mm^2^ area, amyloid plaque size (μm^2^), the ratio of fluorescence intensity of 6E10 to DAPI and the percentage of 6E10 fluorescence intensity in cortical region, (*n* = 15, from three mice per group). *p < 0.05, **p < 0.01, ****p < 0.0001.

Amyloid plaque deposition is one of the main pathological features of AD. To reveal the influence of SV2A on amyloid plaque deposition in the brain tissues of APP/PS1 mice, the frozen sections of brain tissues from the APP/PS1 mice were subsequently subjected to Thioflavin S (TS) staining. The result showed that Aβ plaque deposition in the hippocampal and cortical regions of APP/PS1 mice with SV2A overexpression was significantly reduced when compared to the control group mice, and the quantitative analysis of Aβ plaques showed that the number, the size, and the area of Aβ plaques were significantly reduced (Figure 1a,b), suggesting that SV2A reduced the amyloid plaque deposition in the brain regions of AD mice. In addition, the fluorescence intensity of 6E10 (an Aβ marker) in the brain tissues of the abovementioned two groups of APP/PS1 mice was observed by immunofluorescence, and the results revealed that the number and size of Aβ plaques and 6E10 antibody staining intensity, as well as the fluorescence area of 6E10, was significantly reduced in both the hippocampal (Figure 1c,d) and cortical regions (Figure 1e,f) of the SV2A overexpressed APP/PS1 mice when compared with the control APP/PS1 mice, thereby further indicating that SV2A could significantly reduce the amyloid plaque deposition in APP/PS1 mice.

### 3.2 SV2A Inhibited the Amyloidogenic Processing of APP In Vivo and In Vitro

**FIGURE 2:**
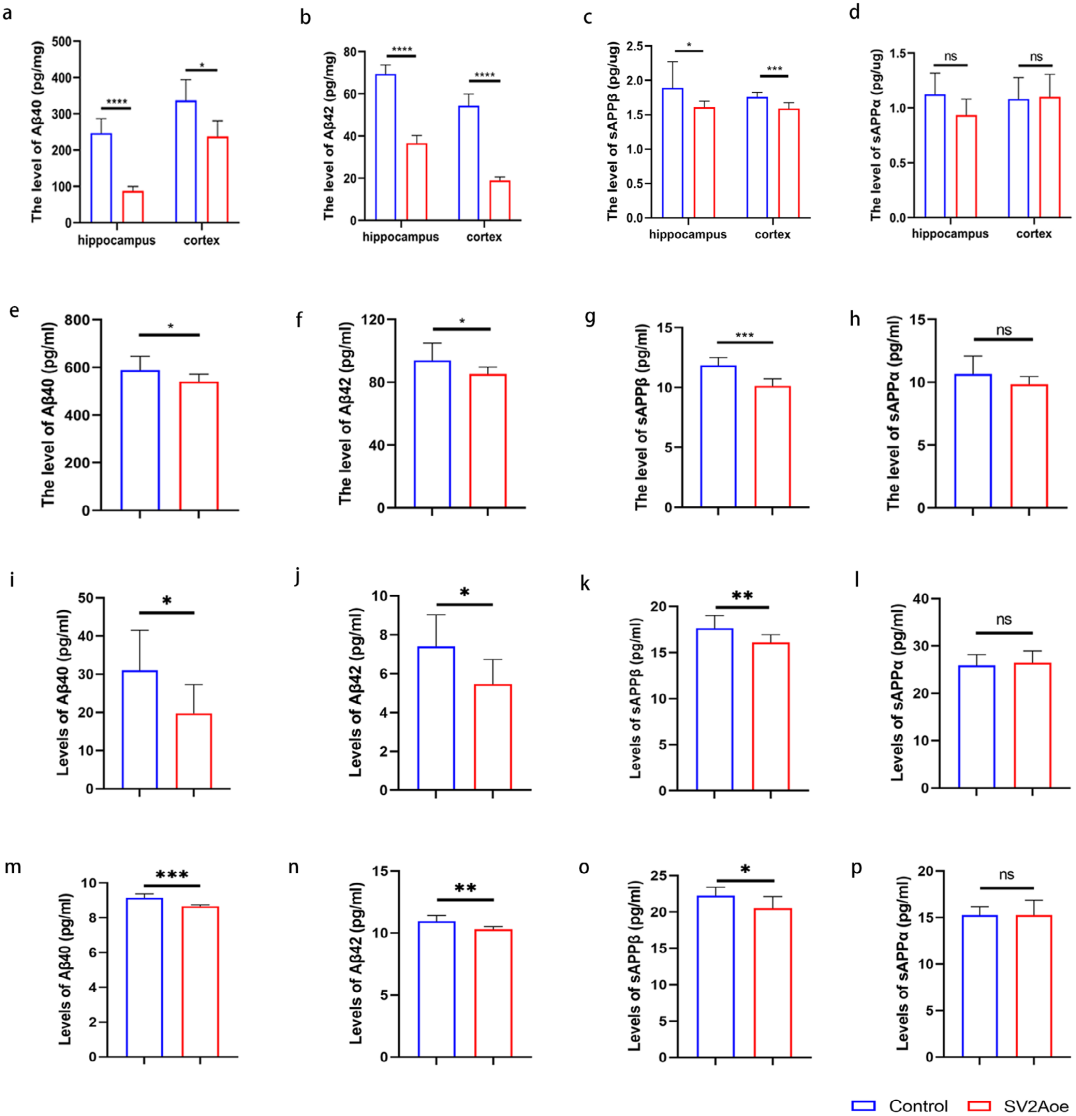
Effect of SV2A on APP degradation in vitro and in vivo. (a–d) Levels of Aβ40, Aβ42, sAPPβ, sAPPα in the hippocampus and cortex of APP/PS1 mice injected with AAV‐SV2Aoe or AAV‐Con. (e–h) Levels of Aβ40, Aβ42, sAPPβ, sAPPα in the serum of APP/PS1 mice injected with AAV‐SV2Aoe or AAV‐Con. (i–l) Levels of Aβ40, Aβ42, sAPPβ, and sAPPα in the supernatants of primary hippocampal neuronal cells infected with SV2Aoe or control lentiviruses. (m–p) Levels of Aβ40, Aβ42, sAPPβ, and sAPPα in the supernatants of SH‐SY5Y‐APP cells transfected with SV2Aoe or control plasmid. Data are presented as the mean ± SD. All dot plots: *t*‐test. *p < 0.05, **p < 0.01, ***p < 0.001, ****p < 0.0001.

The abovementioned studies revealed that SV2A significantly reduced the Aβ plaque deposition in APP/PS1 mice, suggesting that SV2A was involved in APP degradation. Therefore, we next observed the effect of SV2A on APP degradation in APP/PS1 mice. Briefly, the hippocampal and cortical tissues of APP/PS1 mice injected with AAV9‐SV2Aoe or AAV9‐Con were isolated and then homogenized, and the levels of APP and its degradation products in the lysate were further examined, with the result revealing that the levels of Aβ40 (Figure 2a), Aβ42 (Figure 2b), and sAPPβ (Figure 2c) in APP/PS1 mice with SV2A‐overexpression were significantly lower than those in the control group mice, both in the hippocampal and cortical tissues. As for sAPPα, no significant differences were noted between the experimental and control groups in both the hippocampal and cortical tissues (Figure 2d). On the other hand, the levels of APP metabolites in the serum of these two groups of APP/PS1 mice were also measured. The results demonstrated that the serum levels of Aβ40 (Figure 2e), Aβ42 (Figure 2f), and sAPPβ (Figure 2g) in the APP/PS1 mice with SV2A‐overexpression were significantly lower than those in the control group mice, while the levels of sAPPα between the two groups were not statistically significant (Figure 2h).

The above studies revealed that SV2A inhibited the amyloid degradation of APP in vivo. Next, cellular experiments were performed to further validate these findings in vitro. First, a primary hippocampal neuronal cell model of SV2A overexpression was constructed by infection with SV2Aoe lentivirus, and the levels of APP metabolites were detected in the cell supernatant. When compared with the corresponding controls, the upregulation of SV2A significantly decreased the levels of Aβ40, Aβ42, and sAPPβ (Figure 2i–k), while no significant difference was found in the level of sAPPα (Figure 2l). In addition, the SH‐SY5Y‐APP cell model with SV2A overexpression was constructed by transfecting the SV2Aoe plasmid, followed by the detection of APP metabolites. Consistent with the results observed in primary hippocampal neuronal cells, the upregulation of SV2A significantly reduced the level of Aβ42, Aβ40, and sAPPβ (Figure 2m–o) in the supernatant of SH‐SY5Y‐APP cells, while no significant difference in the levels of sAPPα was observed (Figure 2p).

### 3.3 SV2A Was Identified as a Binding Protein of APP

**FIGURE 3:**
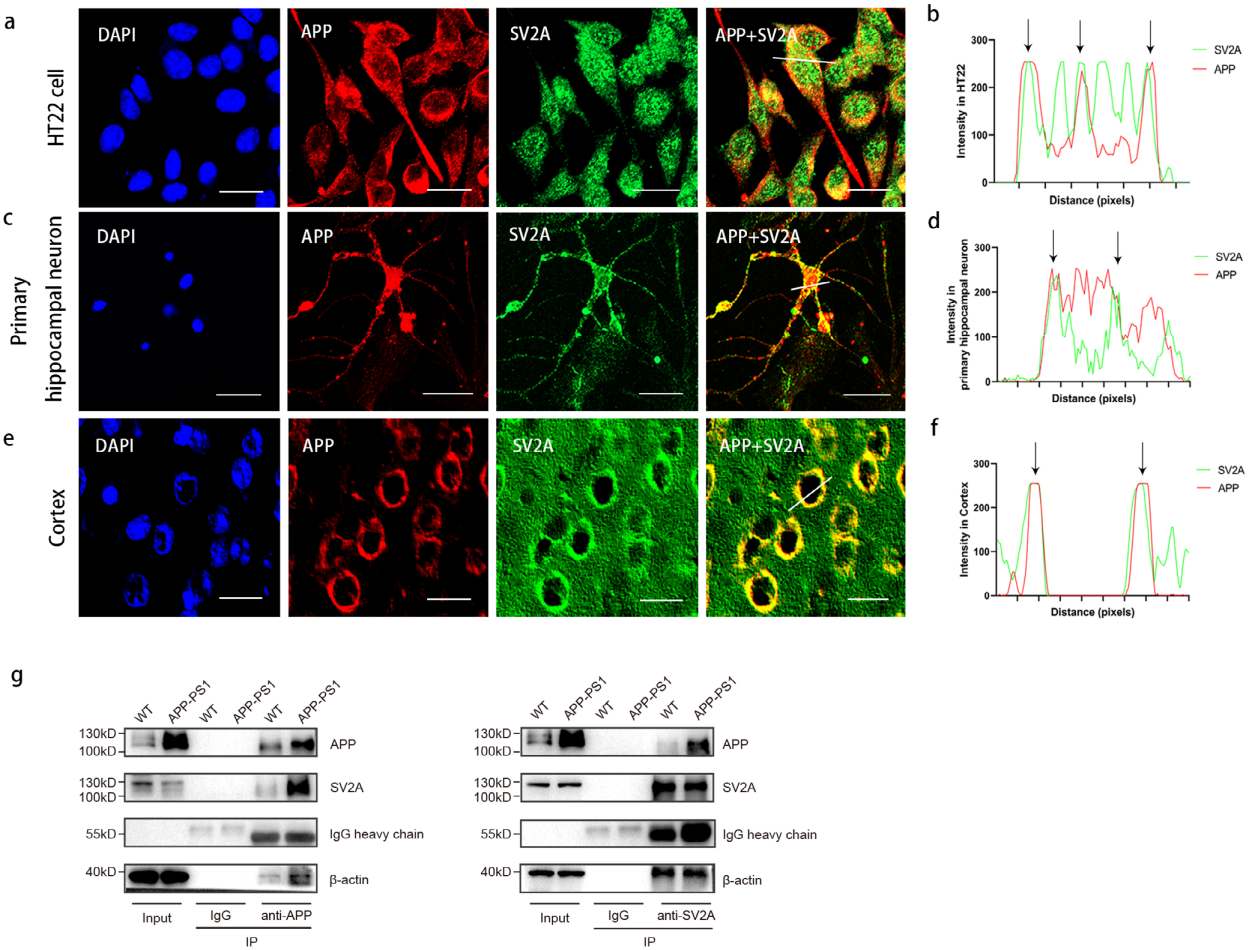
Experiments of SV2A binding with APP. (a) Co‐localization of SV2A and APP in HT22 cells; scale bar: 10 μm. (b) Visualization of SV2A and APP co‐localization in HT22 cells. (c) Co‐localization of SV2A and APP in primary hippocampal neurons isolated from APP/PS1 fetal mice; scale bar: 10 μm. (d) Visualization of SV2A and APP co‐localization in primary hippocampal neurons isolated from APP/PS1 fetal mice. (e) Co‐localization of APP with SV2A in the cortical regions of 3‐month‐old APP/PS1 mice; scale bar: 10 μm. (f) Visualization of SV2A and APP co‐localization in the cortical tissues of APP/PS1 mice. (g) Co‐IP test results of SV2A and APP in brain tissue of wild‐type mice and APP/PS1 mice. Co‐localization analysis was performed by Image J by measuring fluorescence intensity alongside the drawn line. Data are presented as the mean ± SD. All dot plots: *t*‐test.

The abovementioned studies demonstrated that SV2A played an important role in the amyloid degradation of APP, suggesting a potential interaction between the two. To further analyze the interaction between SV2A and APP, co‐localization of the two proteins was first preliminarily observed by immunofluorescence experiments, and the results indicated a significant co‐localization of SV2A and APP in HT22 cells (Figure 3a–b), primary hippocampal neurons (Figure 3c–d), and the cortical region of APP/PS1 mice (Figure 3e–f). To further confirm the interaction between SV2A and APP, CO‐IP experiments were performed in the cortex of 12‐month‐old wild‐type (WT) and APP/PS1 mice. The results showed that SV2A could be immunoprecipitated by anti‐APP antibodies, while APP could be immunoprecipitated by anti‐SV2A antibodies (Figure 3g), suggesting that SV2A was the binding protein of APP.

### 3.4 SV2A Inhibited the Binding of BACE1 to APP

**FIGURE 4:**
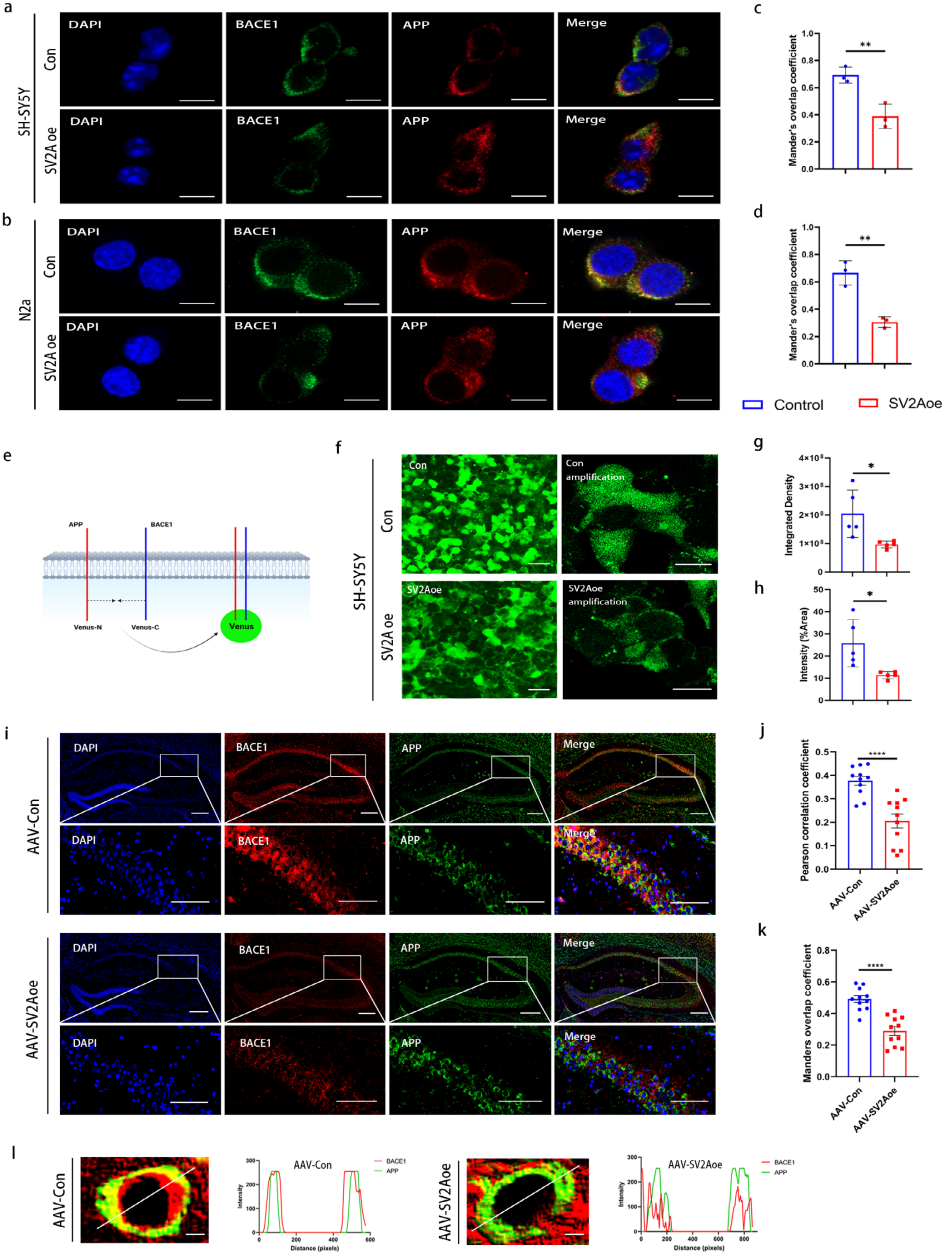
Effect of SV2A overexpression on the binding of BACE1 to APP. (a) Co‐localization of BACE1 with APP in SV2A overexpressed SH‐SY5Y cells; scale bar: 10 μm. (b) Co‐localization of BACE1 with APP in SV2A‐overexpressed N2a cells; scale bar: 10 μm. (c) Quantification of BACE1 co‐localization with APP by Manders overlap coefficient (MOC) in SV2A‐overexpressed SH‐SY5Y cells. (d) Quantification of BACE1 co‐localization with APP by Manders overlap coefficient (MOC) in SV2A‐overexpressed N2a cells. (e) Schematic depiction of bimolecular fluorescence complementation assay (BiFC), wherein APP and BACE1 are tagged with complementary fragments of Venus fluorescent protein (VN and VC, respectively). The reconstitution of Venus fluorescence occurs upon the interaction of APP and BACE1. (f) Representative images of Venus fluorescence in SH‐SY5Y cells transfected with SV2Aoe or control plasmids; scale bar: 10 μm. (g) Integrated density of Venus fluorescence in SH‐SY5Y cells transfected with SV2Aoe or control plasmids. (h) Percentage of area of Venus fluorescence in SH‐SY5Y cells transfected with SV2Aoe or control plasmids. (i) Co‐localization of BACE1 and APP in the hippocampal tissues of APP/PS1 mice injected with AAV‐SV2Aoe or AAV‐Con; scale bar: 50 μm. (j, k) Quantification of BACE1 co‐localization with APP by pearson correlation coefficient (PCC) and manders overlap coefficient (MOC). (l) Co‐localization of BACE1 with APP performed by Image J by measuring fluorescence intensity alongside the drawn line. The co‐localization analysis was performed using Image J software. Data are presented as the mean ± SD. All dot plots: *t*‐test. *p < 0.05, **p < 0.01, ****p < 0.0001.

As the first cleavage enzyme of the APP amyloid metabolic pathway, the binding of BACE1 to APP is the key link to initiate APP amyloid degradation. The above results suggested that SV2A could bind with APP, which prompted us to further investigate the effect of this binding on the induced fit of BACE1 to APP. SH‐SY5Y and N2a cell lines overexpressing SV2A were established separately, and the co‐localization of BACE1 with APP was first observed by immunofluorescence in vitro. The results showed that the degree of co‐localization of BACE1 and APP was reduced, and the Manders overlap coefficient (MOC) was significantly reduced in SV2A‐overexpressed SH‐SY5Y cells or N2a cells when compared with that in the control cells (Figure 4a–d). The binding of BACE1 to APP in the SH‐SY5Y cells transfected with the SV2Aoe plasmid or the control plasmid was further detected by bimolecular fluorescence complementation assay (BiFC), and the related principle is illustrated in Figure 4e. Briefly, APP plasmid (C‐terminally tagged with Venus‐N) and BACE1 plasmid (C‐terminally tagged with Venus‐C) were co‐transfected into SH‐SY5Y cells for 24 h. The binding of BACE1 to the APP protein prompted the combination of Venus‐N and Venus‐C to form an intact Venus protein, whose green fluorescence intensity was directly proportional to the binding capacity of BACE1 to APP. The results of BiFC displayed that the integrated density and the area (%Area) of Venus fluorescence were significantly reduced in SV2A‐overexpressed SH‐SY5Y cells when compared with the control group, which implied that the binding ability of APP to BACE1 was weakened (Figure 4f–h).

The co‐localization of BACE1 with APP in the hippocampal region of 9‐month‐old APP/PS1 mice injected with AAV‐SV2Aoe or AAV‐Con was observed by immunofluorescence to further analyze the effect of SV2A on the binding of BACE1 to APP. The results showed that the co‐localization of BACE1 with APP was reduced in the hippocampal region of SV2A‐overexpressed APP/PS1 mice when compared to the control group (Figure 4i) and that the degree of co‐localization of these two proteins was further assessed by Pearson's correlation coefficient (PCC) and MOC, showing that both PCC (Figure 4j) and MOC (Figure 4k) were significantly reduced in the hippocampal region of APP/PS1 mice overexpressing SV2A when compared to controls. In addition, the visualization of individual cells in the hippocampal region also revealed reduced co‐localization of BACE1 with APP in SV2A‐overexpressed cells (Figure 4l).

These results demonstrated that SV2A inhibited the binding of BACE1 to APP, which could mainly contribute to the downregulation of APP amyloid degradation.

### 3.5 SV2A Reduced the Localization of APP in the Endosomal‐Lysosomal System

Given that the distribution of APP greatly influences its degradation, we next examined the effect of SV2A on APP distribution to elucidate the underlying molecular mechanism.

**FIGURE 5:**
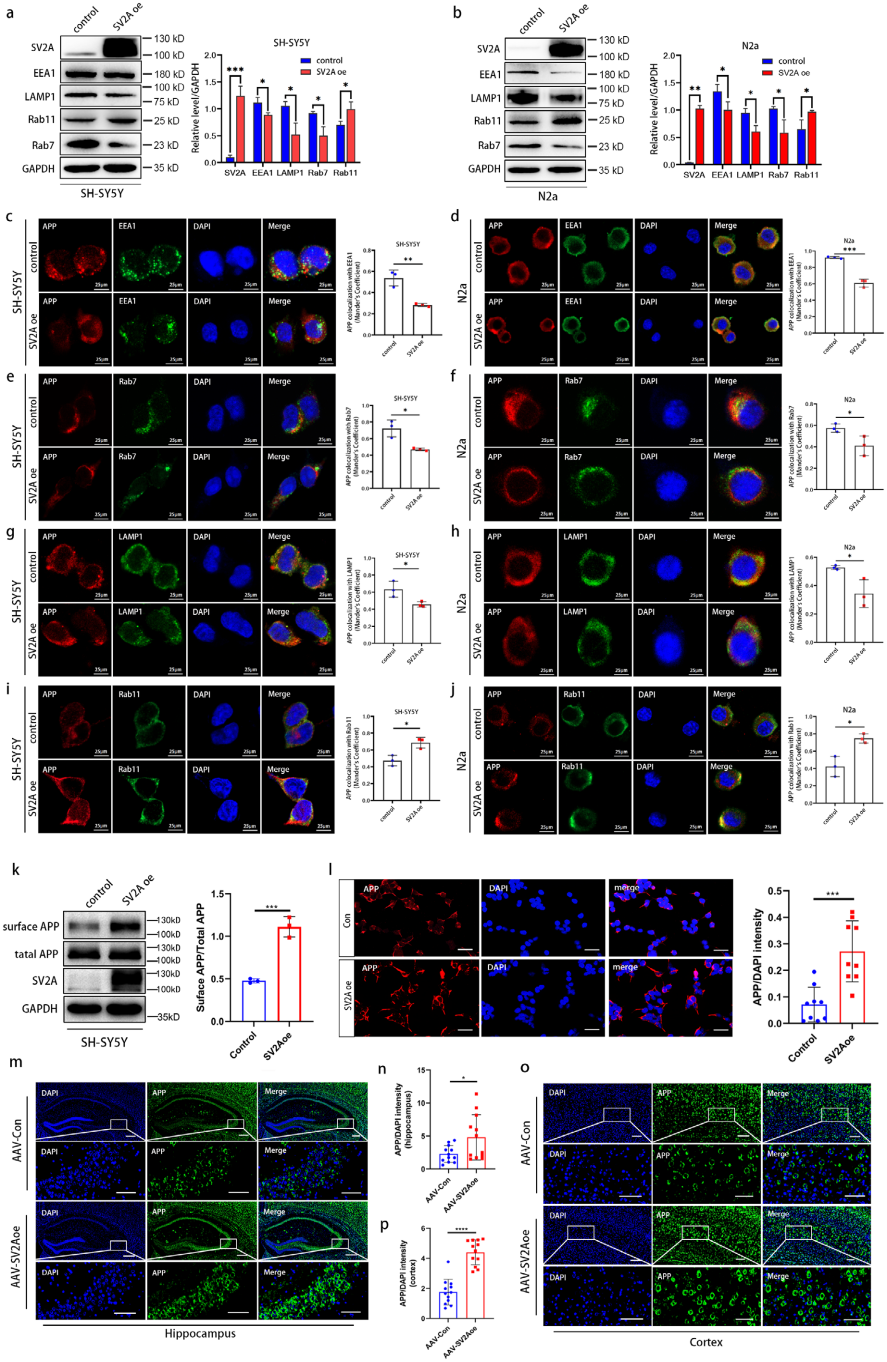
Effect of SV2A overexpression on the distribution of APP. (a, b) Expressions of EEA1, Rab7, Rab11, and LAMP1 in SV2A‐overexpressed SH‐SY5Y cells (N2a cells) and their control cells, as well as the relative expression analysis. (c, d) Fluorescence intensity of EEA1 and APP in SV2A‐overexpressed SH‐SY5Y cells (N2a cells) and the co‐localization analysis of EEA1 and APP. (e, f) Fluorescence intensities of Rab7 and APP in SV2A‐overexpressed SH‐SY5Y cells (N2a cells) and the co‐localization analysis of Rab7 and APP. (g, h) Fluorescence intensity of Rab11 and APP in SV2A‐overexpressed SH‐SY5Y cells (N2a cells) and the co‐localization analysis of Rab11 and APP. (i–j) Fluorescence intensities of LAMP1 and APP in SV2A‐overexpressed SH‐SY5Y cells (N2a cells) and the co‐localization analysis of LAMP1 and APP. (k) Quantification of surface APP expression in SH‐SY5Y cells transfected with SV2Aoe or control plasmids. (l) Quantification of surface APP fluorescence intensity (APP/DAPI) in SH‐SY5Y cells transfected with SV2Aoe or control plasmids, scale bar: 10 μm. (m) Fluorescence level of APP on the cell surface in hippocampal regions of APP/PS1 mice injected with AAV‐SV2A or AAV‐Con; scale bar: 50 μm. (n) Relative fluorescence intensity of APP (APP/DAPI) on the cell surface in the hippocampal regions of APP/PS1 mice injected with AAV‐SV2A or AAV‐Con. (o) Fluorescence level of APP on the cell surface in cortical regions of APP/PS1 mice injected with AAV‐SV2A or AAV‐Con; scale bar: 50 μm. (p) Relative fluorescence intensity of APP (APP/DAPI) on the cell surface in the cortical regions of APP/PS1 mice injected with AAV‐SV2A or AAV‐Con. The protein levels of APP on the cell surface were measured by the cell surface biotin assay. Data are presented as the mean ± SD. All dot plots: *t*‐test. *p < 0.05, **p < 0.01, ***p < 0.001, ****p < 0.0001.

The endo‐lysosomal network is the main location for the amyloid degradation of APP. Therefore, we first explored the effect of SV2A on the distribution of APP in the endo‐lysosomal system. The level of EEA1 (early endosome marker), Rab7 (late endosome marker), Rab11 (recycling endosome marker), and LAMP1 (lysosome marker) in SV2A‐overexpressed SH‐SY5Y cells was first measured by Western blotting. The results showed that the levels of EEA1, Rab7, LAMP1, and their relative expressions were significantly decreased in SV2A‐overexpressed SH‐SY5Y cells when compared to those in the control cells, whereas the levels of Rab11 were significantly increased (Figure 5a); the same results were also detected in N2a cells (Figure 5b). Subsequently, the levels of abovementioned indicators and the co‐localization of these indicators with APP were further observed by immunofluorescence. The results showed that the co‐localization of EEA1 (Figure 5c,d), Rab7 (Figure 5e,f), LAMP1 (Figure 5g,h), and APP was reduced in SV2A‐overexpressed SH‐SY5Y (N2a) cells when compared to that in the control, and the corresponding MOC of APP co‐localized with the above markers was found to be significantly reduced based on the Image J software analysis. However, the co‐localization of APP with Rab11 was increased in SV2A‐overexpressed SH‐SY5Y cells or N2a cells when compared with the control, and the MOC of APP co‐localized with Rab11 was significantly increased, as analyzed by Image J software (Figure 5i,j). These results showed that SV2A‐overexpression reduced the co‐localization of APP with EEA1, Rab7, or LAMP1, whereas it increased the co‐localization of APP with Rab11, which implies that APP is less distributed by SV2A upregulation in early endosomes, late endosomes, and lysosomes, but more distributed in recycling endosomes.

Given that the above research demonstrated SV2A promotes APP localization to recycling endosomes, we next assessed its effect on surface APP distribution using the cell surface biotinylation assay. Briefly, the APP on the cell surface of SV2A‐overexpressed SH‐SY5Y cells was labeled by biotin, respectively, and then the level of labeled APP on the cell surface was detected by Western blotting. The results revealed that the level of APP on the surface of SV2A‐overexpressed SH‐SY5Y cells was significantly increased when compared with that of the control cells and that the ratio of cell surface APP to total APP was also significantly increased (Figure 5k). In addition, the fluorescence level of APP on the cell surface was further observed by immunofluorescence, indicating that the relative fluorescence intensity of APP on the cell surface (APP/DAPI) of SV2A‐overexpressed SH‐SY5Y cells was significantly higher than that of the control cells (Figure 5l). To further validate the abovementioned conclusions, the fluorescence intensity of APP on the cell surface of hippocampal and cortical regions of APP/PS1 mice injected with AAV‐SV2Aoe and AAV‐Con was observed by immunofluorescence, respectively. The results showed that the relative fluorescence intensity of APP (APP/DAPI) on the cell surface of the hippocampal (Figure 5m,n) or the cortical regions (Figure 5o,p) was significantly higher in SV2A‐overexpressed APP/PS1 mice when compared with that in the control mice.

## 4 Discussion

**FIGURE 6:**
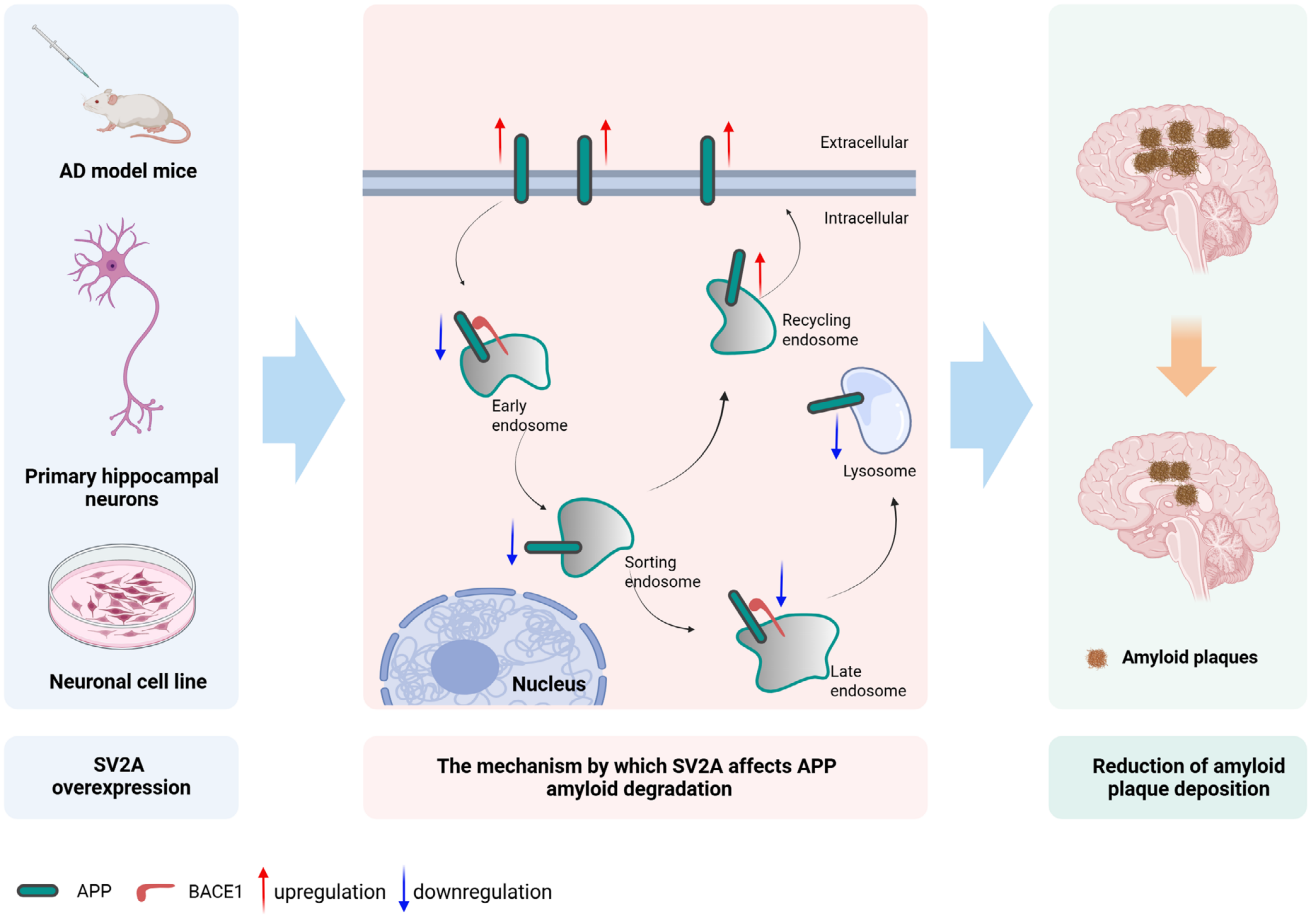
The diagram on the mechanism by which SV2A reduces amyloid plaque deposition. The molecular mechanism by which SV2A affects Aβ deposition is as follows: (1) SV2A overexpression reduces the localization of APP in early endosomes, late endosomes, and lysosomes, while increasing its localization in recycling endosomes and the plasma membrane; (2) as a binding per of APP, SV2A overexpression reduces the level of BACE1‐APP interaction. Through these mechanisms, SV2A overexpression inhibits the amyloidogenic pathway of APP, consequently leading to a reduction in Aβ protein.

In the present study, we revealed the role of SV2A in the pathologic progression of AD through both in vivo and in vitro experiments. Firstly, SV2A overexpression decreased the distribution of APP in the early endosomes, late endosomes, and lysosomes, while increasing its distribution in the recycling endosomes and cell membrane. In addition, as the binding protein of APP, SV2A overexpression inhibited the binding of BACE1 to APP in the endo‐lysosomal network. Through these mechanisms, SV2A overexpression inhibited the amyloid degradation pathway of APP, ultimately leading to a reduction in Aβ protein and amyloid plaques (Figure 6).

SV2A, mainly located in the peripheral sympathetic synapses and presynaptic vesicle terminals, was recognized as the first biomarker reflecting synaptic density in the brain (Holmes et al. 2019). Quite a few studies have shown that SV2A is involved in several neurological conditions, especially as a biomarker evaluated for synaptic loss in the brain of AD by PET Image (Bastin et al. 2020; Chen et al. 2018; Mecca et al. 2022; Venkataraman et al. 2022). In this study, SV2A levels were found to be significantly decreased in postmortem hippocampal and cortex of donors with AD, as well as in CSF and serum of aMCI and AD patients, and their levels were significantly correlated with cognitive scores (Figure S2). The above findings indicated that SV2A could be involved in the occurrence and development of AD. Then, we assessed the cognitive levels of APP/PS1 mice overexpressing SV2A to further explore the effect of SV2A on cognitive ability in AD, and the results showed that SV2A upregulation could significantly improve the cognitive function of AD model mice (Figure S3). SV2A was identified as the specific target of levetiracetam (LEV) (Yamagata et al. 2024). It has been shown that AD model mice demonstrated significant reversal in hippocampal remodeling, behavioral abnormalities, synaptic dysfunction, and learning and memory deficits after long‐term treatment with LEV (Sanchez et al. 2012). Cumbo et al. found that LEV was able to improve cognitive level in AD and aMCI patients (Cumbo and Ligori 2010). Bakker et al. also found that LEV reduced hyperactivity in the hippocampal region of the brain and improved memory in aMCI patients (Bakker et al. 2012). In addition, it has been found that LEV increased SV2A levels in the hippocampal tissue of rats with lipopolysaccharide (LPS)‐induced neuroinflammation (Kasatkina et al. 2022), so the increased cognitive level of SV2A overexpressing AD mice detected in the present study may be one of the molecular mechanisms by which LEV improves cognitive level in AD. In the present study, we further observed that Aβ plaque deposition was significantly reduced in hippocampal and cortical regions of APP/PS1 mice with SV2A overexpression, which may be the important mechanism by which SV2A improved the cognitive level of APP/PS1 mice. We therefore hypothesized that the reduction of SV2A may be a triggering factor for Aβ plaque formation and deposition.

Aβ is produced by APP via the amyloid degradation pathway, implying that SV2A may be involved in this process. APP is first cleaved by BACE1 to start the amyloid degradation pathway, producing sAPPβ and CTF‐β, and then CTF‐β is further cleaved by γ‐secretase to produce Aβ42 and Aβ40, while in non‐amyloid degradation, APP is cleaved by a‐secretase to produce sAPPα and CTF‐α (O'Brien and Wong 2011). In the present study, the overexpression of SV2A resulted in a significant decrease in the levels of Aβ42, Aβ40, and sAPPβ, both in the brain tissue and serum of APP/PS1 mice and in primary hippocampal neurons. However, the levels of sAPPα were not significantly different between the SV2A up‐regulated group and the control group. These results indicated that SV2A inhibited APP amyloid degradation while having no effect on non‐amyloid degradation, which provided a good explanation of the reduction of Aβ plaques in the brain of SV2A‐overexpressed APP/PS1 mice.

These findings indicated that SV2A significantly modulates the amyloidogenic pathway of APP. This prompted further investigation, which identified the key molecular mechanisms central to this regulatory role: the direct interaction between SV2A and APP, the impairment of BACE1‐APP binding, and the influence on APP endosomal‐lysosomal trafficking.

The binding protein of APP is one of the key factors affecting its degradation. In this study, the preliminary screening of APP‐binding proteins was first performed in hippocampal tissues of 9‐month‐old APP/PS1 mice by GST‐pulldown assay combined with Mass spectrometry analysis (Table S1), followed by confirmation of SV2A‐APP interaction in HEK293T cells (Figure S1), HT22 cells, and primary hippocampal neurons and cortical tissues of APP/PS1 mice. The above studies suggested that SV2A formed a complex with APP, and the binding of the two may be the initiating step for SV2A to influence the degradation of APP. Considering the potential for SV2A binding to induce structural changes in APP, this study investigated its effect on the induced fit between BACE1 and APP, which is the crucial first step in the amyloidogenic cleavage of APP within the endosomal‐lysosomal system (Liebsch et al. 2019; Nakamura et al. 2021; Singh et al. 2022; Zhang et al. 2023). Our results revealed that the co‐localization degree of APP and BACE1 in the hippocampal tissues of SV2A‐overexpressed APP/PS1 mice and cells was significantly lower than that in the control groups. In addition, the BiFC results demonstrated the binding ability of APP and BACE1 in SV2A‐overexpressed cells was weakened and that the binding positions of the two proteins tended toward the cell membrane. These results fully confirm that SV2A inhibited the binding of BACE1 and APP, which could be another key mechanism of SV2A inhibiting APP amyloid degradation.

The distribution of APP, both intracellular and extracellular, is a key determinant of its amyloidogenic cleavage. Following endocytosis, APP on the cell surface is targeted to early endosomes and subsequently sorted into three different pathways: (I) a subset of APP is targeted to late endosomes which fuse with lysosomes where APP is degraded (Haass et al. 1992); (II) the undegraded APP is recycled back to the cell surface by recycling pathways (Das et al. 2013); (III) another fraction of APP is transported retrogradely from endosomes back to the TGN (Trans Golgi Network) in a retromer‐mediated pathway (Willnow and Andersen 2013). The endosomal‐lysosomal network, especially in early endosomes and late endosomes, is the primary site where APP underwent amyloid degradation (Dinamarca et al. 2019; Hung and Livesey 2021; Kapadia et al. 2024; Wu et al. 2022), while undegraded APP is recycled to the cell membrane via the recycling pathway. SV2A is critical to vesicle trafficking and exocytosis, neurotransmitter (e.g., GABA and glutamate) transport and release, and the regulation of calcium sensitivity (Bae et al. 2020; Gillespie et al. 2005; Stout et al. 2019). For example, SV2A can be synergistically transported to vesicles with synaptotagmin, and the deletion of SV2A or mutation of its endocytosis motif will reduce the localization of synaptotagmin in vesicles (Yao et al. 2010). In the present study, SV2A inhibited the formation of early endosomes, late endosomes, and the localization of APP therein, and therefore reduced β‐cleavage of APP, which in turn decreased the production of Aβ. In contrast to the results observed in early endosomes, late endosomes, and lysosomes, SV2A promoted both the levels of circulating endosomes and their co‐localization with APP, as well as the distribution of SV2A on the cell surface. Taken together, we hypothesize that SV2A inhibits APP localization in the endosomal‐lysosomal system and promotes its recycling to the cell membrane, thereby inhibiting APP amyloid degradation in endosomes; however, the exact mechanism still needs to be elucidated.

In this study, SV2A was demonstrated to inhibit the pathological progression of AD by downregulating the amyloid degradation of APP. As a binding protein of APP, SV2A reduces the amyloidogenic pathway of APP by inhibiting the binding of BACE1 to APP and regulating the distribution of APP in the cell membrane and endo‐lysosomal network. Therefore, SV2A could be a potential target with promising prospects in the early intervention of AD in the future.

## Author Contributions

P.W. conceived the project and designed the experiments, and part of the paper writing. X.W. contributed to the design and conduct of the experiments and the writing of the article. Q.Z. contributed to part of the experiments and article writing. X.Z. was responsible for part of the experiments and related data collection, and part of the paper writing. J.L. contributed to the experimental technical consultations. J.Z., C.L., Y.C., Q.S., Y.H., Y.W., and M.C. contributed to the data acquisition and analysis. All authors reviewed and revised the manuscript.

## Funding

This study was supported by State Key Program of the National Natural Science Foundation of China, Grant/Award Number: 82030064; National Natural Science Foundation of China, Grant/Award Number: 81871714; Beijing Natural Science Foundation, Grant/Award Number: L246009; HUIZHI Talent Leadership Development Program of Xuanwu Hospital, Grant/Award Number: HZ2021PYLJ023.

## Ethics Statement

This study was approved by the ethics committee of Xuanwu Hospital Capital Medical University, Beijing, China, and was carried out in accordance with the principles of the Declaration of Helsinki.

## Consent

Written informed consent was obtained from all participants or their legal guardians.

## Conflicts of Interest

The authors declare no conflicts of interest.

## Supporting information


**Figure S1:** The Co‐IP results of APP with 11 candidate binding proteins in HEK293T cells. (a–k) The Co‐IP results of APP and candidate proteins in the lysates of HEK293T cells co‐transfected with APP‐myc/Sv2a‐FLAG (a), App‐myc/Adamts7‐FLAG (b), App‐myc/Clu‐FLAG (c), App‐myc/Epn1‐FLAG (d), App‐myc/Hnrnph2‐FLAG (e), App‐myc/Homer1‐FLAG (f), App‐myc/Med23‐FLAG (g), App‐myc/Nefh‐FLAG (h), App‐myc/Sh3gl2‐FLAG (i), App‐myc/Synj1‐FLAG (j) or App‐myc/Vps35‐FLAG (k) for 48–72 h.
**Figure S2:** SV2A levels in AD patients. SV2A expression (in TPM) in postmortem hippocampal (a), entorhinal cortex (b), and temporal cortex (c) samples from donors with AD and cognitively normal controls, as provided by the AlzData database. (d) CSF SV2A levels at different stages of AD and VaD (Con = 45, aMCI = 13, AD = 41, VaD = 13). (e) Correlation of the CSF SV2A levels with MMSE scores. (f) Correlation of the CSF SV2A levels with MOCA scores. (g) Serum SV2A levels at different stages of AD and VaD (Con = 95, aMCI = 80, AD = 145, VaD = 38). (h) Correlation of the serum SV2A level with MMSE scores. (i) Correlation of the serum SV2A level with MOCA scores. Data are presented as the means ±SD. The significance of the between‐group differences was determined using the Mann–Whitney *U*‐test. One‐way ANOVA with Bonferroni post hoc correction was applied to compare the statistical differences between multiple groups. Partial correlation analyses were performed to assess the correlations between biomarkers and cognitive scores by controlling for confounders such as age and sex. *p < 0.05, **p < 0.01, ***p < 0.001, ****p < 0.0001.
**Figure S3:** Cognitive performance in SV2A‐upregulated APP/PS1 mice. (a) Timeline of behavioral experiments performed in APP/PS1 mice injected with AAV‐SV2Aoe or AAV‐Con. (b) Discrimination index of the AAV‐SV2Aoe injected APP/PS1 mice during the test phase NOR. (c) Percentage of total active time of the AAV‐SV2Aoe injected APP/PS1 mice during the training phase of NOR. (d) Escape latency of the AAV‐SV2Aoe injected APP/PS1 mice during the acquisition phase of MWM. (e) Escape latency of the AAV‐SV2Aoe injected APP/PS1 mice entering the original platform region for the first time during the probe phase of MWM. (f) Number of accesses to the platform of the AAV‐SV2Aoe injected APP/PS1 mice during the probe phase of MWM. (g) Swimming speed of the AAV‐SV2Aoe injected APP/PS1 mice during the acquisition phase of MWM. Data were presented as mean ± SD. All dot plots: *t*‐test or one‐way ANOVA test. *p < 0.05, **p < 0.01, ***p < 0.001, ****p < 0.0001.
**Table S1:** Protein identification of APP‐interacting by mass spectrometry analysis.
**Table S2:** Clinical and demographic features of the diagnostic cohorts for CSF and serum SV2A.
**Table S3:** Levels of CSF and serum SV2A in aMCI, AD, and VaD.

## Data Availability

The data that support the findings of this study are available on request from the corresponding author. The data are not publicly available due to privacy or ethical restrictions.

## References

[acel70379-bib-0001] 2023 Alzheimer's Disease Facts and Figures . 2023. “2023 Alzheimer's Disease Facts and Figures.” Alzheimer's & Dementia 19, no. 4: 1598–1695. 10.1002/alz.13016. 36918389

[acel70379-bib-0002] 2024 Alzheimer's Disease Facts and Figures . 2024. “2024 Alzheimer's Disease Facts and Figures.” Alzheimer's & Dementia 20, no. 5: 3708–3821. 10.1002/alz.13809. PMC11095490 38689398

[acel70379-bib-0003] Bae, J. R. , W. Lee , Y. O. Jo , et al. 2020. “Distinct Synaptic Vesicle Recycling in Inhibitory Nerve Terminals Is Coordinated by SV2A.” Progress in Neurobiology 194: 101879. 10.1016/j.pneurobio.2020.101879. 32615146

[acel70379-bib-0004] Bajjalieh, S. M. , G. D. Frantz , J. M. Weimann , S. K. McConnell , and R. H. Scheller . 1994. “Differential Expression of Synaptic Vesicle Protein 2 (SV2) Isoforms.” Journal of Neuroscience 14, no. 9: 5223–5235. 10.1523/JNEUROSCI.14-09-05223.1994. 8083732 PMC6577109

[acel70379-bib-0005] Bakker, A. , G. L. Krauss , M. S. Albert , et al. 2012. “Reduction of Hippocampal Hyperactivity Improves Cognition in Amnestic Mild Cognitive Impairment.” Neuron 74, no. 3: 467–474. 10.1016/j.neuron.2012.03.023. 22578498 PMC3351697

[acel70379-bib-0006] Bastin, C. , M. A. Bahri , F. Meyer , et al. 2020. “In Vivo Imaging of Synaptic Loss in Alzheimer's Disease With [18F]UCB‐H Positron Emission Tomography.” European Journal of Nuclear Medicine and Molecular Imaging 47, no. 2: 390–402. 10.1007/s00259-019-04461-x. 31468182

[acel70379-bib-0007] Calvo‐Rodriguez, M. , E. K. Kharitonova , A. C. Snyder , et al. 2024. “Real‐Time Imaging of Mitochondrial Redox Reveals Increased Mitochondrial Oxidative Stress Associated With Amyloid Beta Aggregates In Vivo in a Mouse Model of Alzheimer's Disease.” Molecular Neurodegeneration 19, no. 1: 6. 10.1186/s13024-024-00702-2. 38238819 PMC10797952

[acel70379-bib-0008] Chen, M. K. , A. P. Mecca , M. Naganawa , et al. 2018. “Assessing Synaptic Density in Alzheimer Disease With Synaptic Vesicle Glycoprotein 2A Positron Emission Tomographic Imaging.” JAMA Neurology 75, no. 10: 1215–1224. 10.1001/jamaneurol.2018.1836. 30014145 PMC6233853

[acel70379-bib-0009] Choi, G. E. , J. Y. Park , M. R. Park , J. H. Yoon , and H. J. Han . 2023. “Glucocorticoid Enhances presenilin1‐Dependent Abeta Production at ER'S Mitochondrial‐Associated Membrane by Downregulating Rer1 in Neuronal Cells.” Redox Biology 65: 102821. 10.1016/j.redox.2023.102821. 37494768 PMC10382667

[acel70379-bib-0010] Cumbo, E. , and L. D. Ligori . 2010. “Levetiracetam, Lamotrigine, and Phenobarbital in Patients With Epileptic Seizures and Alzheimer's Disease.” Epilepsy & Behavior 17, no. 4: 461–466. 10.1016/j.yebeh.2010.01.015. 20188634

[acel70379-bib-0011] Das, U. , D. A. Scott , A. Ganguly , E. H. Koo , Y. Tang , and S. Roy . 2013. “Activity‐Induced Convergence of APP and BACE‐1 in Acidic Microdomains via an Endocytosis‐Dependent Pathway.” Neuron 79, no. 3: 447–460. 10.1016/j.neuron.2013.05.035. 23931995 PMC3741682

[acel70379-bib-0012] Dhillon, S. 2021. “Aducanumab: First Approval.” Drugs 81, no. 12: 1437–1443. 10.1007/s40265-021-01569-z. 34324167

[acel70379-bib-0013] Dinamarca, M. C. , A. Raveh , A. Schneider , et al. 2019. “Complex Formation of APP With GABA(B) Receptors Links Axonal Trafficking to Amyloidogenic Processing.” Nature Communications 10, no. 1: 1331. 10.1038/s41467-019-09164-3. PMC6430795 30902970

[acel70379-bib-0014] Dubois, B. , N. Villain , G. B. Frisoni , et al. 2021. “Clinical Diagnosis of Alzheimer's Disease: Recommendations of the International Working Group.” Lancet Neurology 20, no. 6: 484–496. 10.1016/S1474-4422(21)00066-1. 33933186 PMC8339877

[acel70379-bib-0015] Gauthier, S. , B. Reisberg , M. Zaudig , et al. 2006. “Mild Cognitive Impairment.” Lancet (London, England) 367, no. 9518: 1262–1270. 10.1016/S0140-6736(06)68542-5. 16631882

[acel70379-bib-0016] Gillespie, D. C. , G. Kim , and K. Kandler . 2005. “Inhibitory Synapses in the Developing Auditory System Are Glutamatergic.” Nature Neuroscience 8, no. 3: 332–338. 10.1038/nn1397. 15746915

[acel70379-bib-0017] Haass, C. , C. Kaether , G. Thinakaran , and S. Sisodia . 2012. “Trafficking and Proteolytic Processing of APP.” Cold Spring Harbor Perspectives in Medicine 2, no. 5: a006270. 10.1101/cshperspect.a006270. 22553493 PMC3331683

[acel70379-bib-0018] Haass, C. , E. H. Koo , A. Mellon , A. Y. Hung , and D. J. Selkoe . 1992. “Targeting of Cell‐Surface Beta‐Amyloid Precursor Protein to Lysosomes ‐ Alternative Processing Into Amyloid‐Bearing Fragments.” Nature 357, no. 6378: 500–503. 10.1038/357500a0. 1608449

[acel70379-bib-0019] Holmes, S. E. , D. Scheinost , S. J. Finnema , et al. 2019. “Lower Synaptic Density Is Associated With Depression Severity and Network Alterations.” Nature Communications 10: 1529. 10.1038/s41467-019-09562-7. PMC6449365 30948709

[acel70379-bib-0020] Hung, C. , and F. J. Livesey . 2021. “Endolysosome and Autophagy Dysfunction in Alzheimer Disease.” Autophagy 17, no. 11: 3882–3883. 10.1080/15548627.2021.1963630. 34429033 PMC8632268

[acel70379-bib-0021] Jansen, W. J. , R. Ossenkoppele , D. L. Knol , et al. 2015. “Prevalence of Cerebral Amyloid Pathology in Persons Without Dementia A Meta‐Analysis.” JAMA 313, no. 19: 1924–1938. 10.1001/jama.2015.4668. 25988462 PMC4486209

[acel70379-bib-0022] Janz, R. , Y. Goda , M. Geppert , M. Missler , and T. C. Südhof . 1999. “SV2A and SV2B Function as Redundant ca Regulators in Neurotransmitter Release.” Neuron 24, no. 4: 1003–1016. 10.1016/S0896-6273(00)81046-6. 10624962

[acel70379-bib-0023] Kapadia, A. , S. Theil , S. Opitz , et al. 2024. “Phosphorylation‐State Dependent Intraneuronal Sorting of Abeta Differentially Impairs Autophagy and the Endo‐Lysosomal System.” Autophagy 20, no. 1: 166–187. 10.1080/15548627.2023.2252300. 37642583 PMC10761119

[acel70379-bib-0024] Karran, E. , and B. De Strooper . 2022. “The Amyloid Hypothesis in Alzheimer Disease: New Insights From New Therapeutics.” Nature Reviews Drug Discovery 21, no. 4: 306–318. 10.1038/s41573-022-00391-w. 35177833

[acel70379-bib-0025] Kasatkina, L. A. , V. P. Gumenyuk , O. O. Lisakovska , and I. O. Trikash . 2022. “Targeting Hippocampal Amyloidogenesis With SV2A Protein Modulator Levetiracetam.” Biochemical Pharmacology 197: 114927. 10.1016/j.bcp.2022.114927. 35065023

[acel70379-bib-0026] Kuhn, A. J. , K. Chan , M. Sajimon , et al. 2024. “Amyloid‐Alpha Peptide Formed Through Alternative Processing of the Amyloid Precursor Protein Attenuates Alzheimer's Amyloid‐Beta Toxicity via Cross‐Chaperoning.” Journal of the American Chemical Society 146, no. 4: 2634–2645. 10.1021/jacs.3c11511. 38236059

[acel70379-bib-0027] Kumar, R. , T. le Marchand , L. Adam , et al. 2024. “Identification of Potential Aggregation Hotspots on Aβ42 Fibrils Blocked by the Anti‐Amyloid Chaperone‐Like BRICHOS Domain.” Nature Communications 15, no. 1: 965. 10.1038/s41467-024-45192-4. PMC10834949 38302480

[acel70379-bib-0028] Liebsch, F. , L. Kulic , C. Teunissen , et al. 2019. “Aβ34 Is a BACE1‐Derived Degradation Intermediate Associated With Amyloid Clearance and Alzheimer's Disease Progression.” Nature Communications 10, no. 1: 2240. 10.1038/s41467-019-10152-w. PMC6527709 31110178

[acel70379-bib-0029] Liu, Z. , P. G. Lee , N. Krez , et al. 2023. “Structural Basis for Botulinum Neurotoxin E Recognition of Synaptic Vesicle Protein 2.” Nature Communications 14, no. 1: 2338. 10.1038/s41467-023-37860-8. PMC10125960 37095076

[acel70379-bib-0030] McDade, E. , and R. J. Bateman . 2017. “Stop Alzheimer's Before It Starts.” Nature 547, no. 7662: 153–155. 10.1038/547153a. 28703214

[acel70379-bib-0031] McKhann, G. M. , D. S. Knopman , H. Chertkow , et al. 2011. “The Diagnosis of Dementia due to Alzheimer's Disease: Recommendations From the National Institute on Aging‐Alzheimer's Association Workgroups on Diagnostic Guidelines for Alzheimer's Disease.” Alzheimer's & Dementia 7, no. 3: 263–269. 10.1016/j.jalz.2011.03.005. PMC3312024 21514250

[acel70379-bib-0032] Mecca, A. P. , R. S. O'Dell , E. S. Sharp , et al. 2022. “Synaptic Density and Cognitive Performance in Alzheimer's Disease: A PET Imaging Study With [(11) C]UCB‐J.” Alzheimer's & Dementia 18, no. 12: 2527–2536. 10.1002/alz.12582. PMC9381645 35174954

[acel70379-bib-0033] Mohanty, R. , D. Ferreira , S. Frerich , et al. 2022. “Neuropathologic Features of Antemortem Atrophy‐Based Subtypes of Alzheimer Disease.” Neurology 99, no. 4: e323–e333. 10.1212/WNL.0000000000200573. 35609990 PMC9421777

[acel70379-bib-0034] Montenegro, P. , P. Chen , J. Kang , S. H. Lee , S. Leone , and J. Shen . 2023. “Human Presenilin‐1 Delivered by AAV9 Rescues Impaired Gamma‐Secretase Activity, Memory Deficits, and Neurodegeneration in Psen Mutant Mice.” Proceedings of the National Academy of Sciences of the United States of America 120, no. 42: e2306714120. 10.1073/pnas.2306714120. 37816062 PMC10589670

[acel70379-bib-0035] Musardo, S. , S. Therin , S. Pelucchi , et al. 2022. “The Development of ADAM10 Endocytosis Inhibitors for the Treatment of Alzheimer's Disease.” Molecular Therapy 30, no. 7: 2474–2490. 10.1016/j.ymthe.2022.03.024. 35390543 PMC9263258

[acel70379-bib-0036] Nakamura, M. , Y. Li , B. R. Choi , et al. 2021. “GDE2‐RECK Controls ADAM10 Alpha‐Secretase‐Mediated Cleavage of Amyloid Precursor Protein.” Science Translational Medicine 13, no. 585: abe6178. 10.1126/scitranslmed.abe6178. PMC8085789 33731436

[acel70379-bib-0037] Ng, B. , J. Vowles , F. Bertherat , et al. 2024. “Tau Depletion in Human Neurons Mitigates Abeta‐Driven Toxicity.” Molecular Psychiatry 29, no. 7: 2009–2020. 10.1038/s41380-024-02463-2. 38361127 PMC11408257

[acel70379-bib-0038] O'Brien, R. J. , and P. C. Wong . 2011. “Amyloid Precursor Protein Processing and Alzheimer's Disease.” Annual Review of Neuroscience 34: 185–204. 10.1146/annurev-neuro-061010-113613. PMC3174086 21456963

[acel70379-bib-0039] O'Dell, R. S. , A. P. Mecca , M. K. Chen , et al. 2021. “Association of Abeta Deposition and Regional Synaptic Density in Early Alzheimer's Disease: A PET Imaging Study With [(11)C]UCB‐J.” Alzheimer's Research & Therapy 13, no. 1: 11. 10.1186/s13195-020-00742-y. PMC7786921 33402201

[acel70379-bib-0040] Onwordi, E. C. , T. Whitehurst , E. Shatalina , et al. 2024. “Synaptic Terminal Density Early in the Course of Schizophrenia: An In Vivo UCB‐J Positron Emission Tomographic Imaging Study of SV2A.” Biological Psychiatry 95, no. 7: 639–646. 10.1016/j.biopsych.2023.05.022. 37330164 PMC10923626

[acel70379-bib-0041] Roman, G. C. , T. K. Tatemichi , T. Erkinjuntti , et al. 1993. “Vascular Dementia: Diagnostic Criteria for Research Studies. Report of the NINDS‐AIREN International Workshop.” Neurology 43, no. 2: 250–260. 10.1212/wnl.43.2.250. 8094895

[acel70379-bib-0042] Sanchez, P. E. , L. Zhu , L. Verret , et al. 2012. “Levetiracetam Suppresses Neuronal Network Dysfunction and Reverses Synaptic and Cognitive Deficits in an Alzheimer's Disease Model.” Proceedings of the National Academy of Sciences of the United States of America 109, no. 42: E2895–E2903. 10.1073/pnas.1121081109. 22869752 PMC3479491

[acel70379-bib-0043] Senapati, S. , K. Tripathi , K. Awad , and S. Rahimipour . 2024. “Multifunctional Liposomes Targeting Amyloid‐Beta Oligomers for Early Diagnosis and Therapy of Alzheimer's Disease.” Small (Weinheim an der Bergstrasse, Germany) 20: e2311670. 10.1002/smll.202311670. 38461531

[acel70379-bib-0044] Serneels, L. , D. T'Syen , L. Perez‐Benito , T. Theys , M. G. Holt , and B. de Strooper . 2020. “Modeling the Beta‐Secretase Cleavage Site and Humanizing Amyloid‐Beta Precursor Protein in Rat and Mouse to Study Alzheimer's Disease.” Molecular Neurodegeneration 15, no. 1: 60. 10.1186/s13024-020-00399-z. 33076948 PMC7574558

[acel70379-bib-0045] Singh, N. , B. Das , J. Zhou , X. Hu , and R. Yan . 2022. “Targeted BACE‐1 Inhibition in Microglia Enhances Amyloid Clearance and Improved Cognitive Performance.” Science Advances 8, no. 29: eabo3610. 10.1126/sciadv.abo3610. 35857844 PMC9299535

[acel70379-bib-0046] Stout, K. A. , A. R. Dunn , C. Hoffman , and G. W. Miller . 2019. “The Synaptic Vesicle Glycoprotein 2: Structure, Function, and Disease Relevance.” ACS Chemical Neuroscience 10, no. 9: 3927–3938. 10.1021/acschemneuro.9b00351. 31394034 PMC11562936

[acel70379-bib-0047] Tao, Q. Q. , X. Cai , Y. Y. Xue , et al. 2024. “Alzheimer's Disease Early Diagnostic and Staging Biomarkers Revealed by Large‐Scale Cerebrospinal Fluid and Serum Proteomic Profiling.” Innovation 5, no. 1: 100544. 10.1016/j.xinn.2023.100544. 38235188 PMC10794110

[acel70379-bib-0048] van Dyck, C. H. , C. J. Swanson , P. Aisen , et al. 2023. “Lecanemab in Early Alzheimer's Disease.” New England Journal of Medicine 388, no. 1: 9–21. 10.1056/NEJMoa2212948. 36449413

[acel70379-bib-0049] Venkataraman, A. V. , A. Mansur , G. Rizzo , et al. 2022. “Widespread Cell Stress and Mitochondrial Dysfunction Occur in Patients With Early Alzheimer's Disease.” Science Translational Medicine 14, no. 658: eabk1051. 10.1126/scitranslmed.abk1051. 35976998

[acel70379-bib-0050] Vilella, A. , M. Bodria , B. Papotti , et al. 2024. “PCSK9 Ablation Attenuates Abeta Pathology, Neuroinflammation and Cognitive Dysfunctions in 5XFAD Mice.” Brain, Behavior, and Immunity 115: 517–534. 10.1016/j.bbi.2023.11.008. 37967665

[acel70379-bib-0051] Wang, B. , X. Pan , I. T. Teng , et al. 2024. “Functional Selection of Tau Oligomerization‐Inhibiting Aptamers.” Angewandte Chemie (International Ed. In English) 63, no. 18: e202402007. 10.1002/anie.202402007. 38407551 PMC12498309

[acel70379-bib-0052] Whiteside, D. J. , N. Holland , K. A. Tsvetanov , et al. 2023. “Synaptic Density Affects Clinical Severity via Network Dysfunction in Syndromes Associated With Frontotemporal Lobar Degeneration.” Nature Communications 14, no. 1: 8458. 10.1038/s41467-023-44307-7. PMC10730886 38114493

[acel70379-bib-0053] Willnow, T. E. , and O. M. Andersen . 2013. “Sorting Receptor SORLA–a Trafficking Path to Avoid Alzheimer Disease.” Journal of Cell Science 126, no. 13: 2751–2760. 10.1242/jcs.125393. 23813966

[acel70379-bib-0054] Wu, C. I. , E. A. Vinton , R. V. Pearse 2nd , et al. 2022. “APP and DYRK1A Regulate Axonal and Synaptic Vesicle Protein Networks and Mediate Alzheimer's Pathology in Trisomy 21 Neurons.” Molecular Psychiatry 27, no. 4: 1970–1989. 10.1038/s41380-022-01454-5. 35194165 PMC9133025

[acel70379-bib-0055] Yamagata, A. , K. Ito , T. Suzuki , N. Dohmae , T. Terada , and M. Shirouzu . 2024. “Structural Basis for Antiepileptic Drugs and Botulinum Neurotoxin Recognition of SV2A.” Nature Communications 15, no. 1: 3027. 10.1038/s41467-024-47322-4. PMC11026379 38637505

[acel70379-bib-0056] Yao, J. , A. Nowack , P. Kensel‐Hammes , R. G. Gardner , and S. M. Bajjalieh . 2010. “Cotrafficking of SV2 and Synaptotagmin at the Synapse.” Journal of Neuroscience 30, no. 16: 5569–5578. 10.1523/Jneurosci.4781-09.2010. 20410110 PMC2866018

[acel70379-bib-0057] Zhang, J. , F. Cai , R. B. Lu , et al. 2023. “CNTNAP2 Intracellular Domain (CICD) Generated by γ‐Secretase Cleavage Improves Autism‐Related Behaviors.” Signal Transduction and Targeted Therapy 8, no. 1: 219. 10.1038/s41392-023-01431-6. 37271769 PMC10239753

[acel70379-bib-0058] Zhou, J. , N. Singh , J. Galske , J. Hudobenko , X. Hu , and R. Yan . 2023. “BACE1 Regulates Expression of Clusterin in Astrocytes for Enhancing Clearance of β‐Amyloid Peptides.” Molecular Neurodegeneration 18, no. 1: 31. 10.1186/s13024-023-00611-w. 37143090 PMC10161466

[acel70379-bib-0059] Zhu, Y. , R. Huang , D. Wang , et al. 2023. “EVs‐Mediated Delivery of CB2 Receptor Agonist for Alzheimer's Disease Therapy.” Asian Journal of Pharmaceutical Sciences 18, no. 4: 100835. 10.1016/j.ajps.2023.100835. 37645682 PMC10460952

[acel70379-bib-0060] Zhuravleva, V. , J. Vaz‐Silva , M. Zhu , et al. 2021. “Rab35 and Glucocorticoids Regulate APP and BACE1 Trafficking to Modulate Aβ Production.” Cell Death & Disease 12, no. 12: 1137. 10.1038/s41419-021-04433-w. 34876559 PMC8651661

