# Supplementary material for: Synaptic Vesicle Glycoprotein 2A Suppresses Amyloidogenesis Beyond Its Synaptic Role: A Novel Mechanism Disrupting BACE1 Binding and Altering APP Localization
Source: Aging Cell. 2026 Jan 11;25(2):e70379. doi: 10.1111/acel.70379 (PMC12793060; doi:10.1111/acel.70379)
Supplement: Supplementary file 1 — Figure S1: The Co‐IP results of APP with 11 candidate binding proteins in HEK293T cells. (a–k) The Co‐IP results of APP and candidate proteins in the lysates of HEK293T cells co‐transfected with APP‐myc/Sv2a‐FLAG (a), App‐myc/Adamts7‐FLAG (b), App‐myc/Clu‐FLAG (c), App‐myc/Epn1‐FLAG (d), App‐myc/Hnrnph2‐FLAG (e), App‐myc/Homer1‐FLAG (f), App‐myc/Med23‐FLAG (g), App‐myc/Nefh‐FLAG (h), App‐myc/Sh3gl2‐FLAG (i), App‐myc/Synj1‐FLAG (j) or App‐myc/Vps35‐FLAG (k) for 48–72 h. Figure S2: SV2A levels in AD patients. SV2A expression (in TPM) in postmortem hippocampal (a), entorhinal cortex (b), and temporal cortex (c) samples from donors with AD and cognitively normal controls, as provided by the AlzData database. (d) CSF SV2A levels at different stages of AD and VaD (Con = 45, aMCI = 13, AD = 41, VaD = 13). (e) Correlation of the CSF SV2A levels with MMSE scores. (f) Correlation of the CSF SV2A levels with MOCA scores. (g) Serum SV2A levels at different stages of AD and VaD (Con = 95, aMCI = 80, AD = 145, VaD = 38). (h) Correlation of the serum SV2A level with MMSE scores. (i) Correlation of the serum SV2A level with MOCA scores. Data are presented as the means ±SD. The significance of the between‐group differences was determined using the Mann–Whitney U‐test. One‐way ANOVA with Bonferroni post hoc correction was applied to compare the statistical differences between multiple groups. Partial correlation analyses were performed to assess the correlations between biomarkers and cognitive scores by controlling for confounders such as age and sex. *p < 0.05, **p < 0.01, ***p < 0.001, ****p < 0.0001. Figure S3: Cognitive performance in SV2A‐upregulated APP/PS1 mice. (a) Timeline of behavioral experiments performed in APP/PS1 mice injected with AAV‐SV2Aoe or AAV‐Con. (b) Discrimination index of the AAV‐SV2Aoe injected APP/PS1 mice during the test phase NOR. (c) Percentage of total active time of the AAV‐SV2Aoe injected APP/PS1 mice during the training phase of NOR. (d) Escape late [file ACEL-25-e70379-s001.docx]

**Synaptic vesicle glycoprotein 2A suppresses amyloidogenesis beyond its synaptic role: a novel mechanism disrupting BACE1 binding and altering APP localization**

Xiaoling Wang, Qian Zhang, Xiaomin Zhang, Jing Liu, Jingjing Zhang, Congcong Liu, Yuting Cui, Qiao Song, Yuli Hou, Yaqi Wang, Min Cao and Peichang Wang^*^

**1. Results**

**1.1 Screening and identification of APP-interacting proteins**

APP-binding proteins are key regulators of its degradation. To assess the native APP interactome in brain, the hippocampal proteins of 9-month-old APP/PS1 mice were prepared. We next carried out a GST pulldown assay in mouse hippocampal lysates with GST-labeled APP to identify potential APP associated proteins. Mass spectrometry analysis identified a variety of proteins. We compared the unique peptides and peptide-spectrum matches (PSMs) of the identified proteins from different samples and screened 11 proteins that were most likely to interact with APP (Table S1).

To further verify whether the identified proteins interact with APP, we performed Co-IP experiments *in* *vitro*. HEK293T cells were co-transfected with App-myc/Adamts7-FLAG, App-myc/Sv2a-FLAG, App-myc/Homer1-FLAG, App-myc/Sh3gl2-FLAG, App-myc/Synj1-FLAG, App-myc/Clu-FLAG, App-myc/Vps35-FLAG, App-myc/Nefh-FLAG, App-myc/Hnrnph2-FLAG, App-myc/Epn1-FLAG or App-myc/Med23-FLAG, lysed and then incubated with anti-myc antibodies or IgG, respectively. The results suggested that only SV2A interacted with APP among the 11 candidate proteins in HEK293T cells (Fig. S1).

**1.2 SV2A levels were significantly decreased in AD patients**

To investigate the expression of SV2A during the progression of AD. We first analyzed SV2A expression (in TPM) in postmortem entorhinal cortex, hippocampal, and temporal cortex samples from donors with AD and cognitively normal controls, as provided by the AlzData database. Statistical analysis demonstrated a significant reduction in SV2A expression in AD patients relative to controls across all three regions: hippocampus (*p* < 0.0001), entorhinal cortex (*p* < 0.0001), and temporal cortex (*p* = 0.0002) (Fig. S2a-c).

To analyze the alteration of SV2A levels in the body fluids of AD patients, CSF SV2A levels in aMCI, AD patients, and age-matched controls were first measured by the Simoa technology platform. The results revealed that the CSF SV2A levels were significantly decreased in both aMCI and AD than in the controls, and their levels gradually decreased with the progression of AD. In addition, the levels of CSF SV2A in other types of dementia were examined to reveal no significant difference in the VaD patients relative to the control patients. However, the CSF SV2A levels in AD patients were significantly lower than those in VaD patients (Fig. S2d). Correlational analysis between CSF SV2A and cognitive scale scores was performed to reveal that CSF SV2A were significantly and positively correlated with MMSE (r = 0.2924, *p* = 0.0319) (Fig. S2e) and MOCA scores (r = 0.2726, *p* = 0.0462) (Fig. S2f). The alteration of SV2A levels in the serum of aMCI, AD, and age-matched healthy controls was also measured by using the Simoa method. Consistent with the trend of CSF SV2A, the levels of serum SV2A were significantly decreased in both aMCI and AD relative to that in the control group, and its level in AD was significantly lower than that in aMCI. The level of serum SV2A was not significantly different in VaD patients when compared to that in controls. However, the serum SV2A levels were significantly lower in AD patients than in VaD patients (Fig. S2g). In addition, correlation analyses showed a significant positive correlation between the serum SV2A levels and cognitive scores (MMSE: r = 0.2837, *p* < 0.0001; MOCA: r = 0.2373, *p* = 0.0003) (Fig. S2h–i). The abovementioned data are presented in Table S2 and Table S3, and all data were adjusted for age and sex.

**1.3 SV2A significantly improved cognitive performance in APP/PS1 mice**

The abovementioned results preliminarily revealed the decrease of SV2A in AD. To further analyze the role of SV2A in the development of AD, the effects of SV2A on cognitive performance in APP/PS1 mice were assessed. Briefly, AAV9-SV2Aoe and AAV9-Con were injected into the bilateral hippocampal areas of 9-month-old APP/PS1 mice as the experimental and control groups, respectively. The schedule of behavioral experiments is shown in Fig. S3a. New object recognition experiments (NOR) were first performed 5 weeks after the injection. The results revealed that SV2A overexpressing APP/PS1 mice had a significantly higher discrimination index for novel objects than control mice during the test phase (Fig. S3b) and that the activity time of APP/PS1 mice with SV2A-overexpression was significantly lower than that of the control mice in the familiar phase of NOR (Fig. S3c), implying that SV2A could significantly improve the cognitive performance in APP/PS1 mice.

To further assess the effect of SV2A on the cognitive performance of APP/PS1 mice, the Morris water maze (MWM) experiment was performed. The escape latencies during the acquisition phase were first analyzed, and the results revealed that the escape latency of SV2A-overexpressing APP/PS1 mice was significantly shorter when compared to that of the control group mice, and the difference between the two groups was statistically significant on the fourth and fifth day (Fig. S3d). In the probe phase, SV2A-overexpressing APP/PS1 mice had a significantly shorter escape latency for the first entrance to the original platform region (Fig. S3e) and a significantly increased number of entrances (Fig. S3f) when compared to the control APP/PS1 mice. In addition, the swimming speed of SV2A-overexpressing APP/PS1 mice and the control group during the acquisition phase was analyzed to display no statistical difference between the two groups (Fig. S3g), suggesting that SV2A did not affect the locomotor
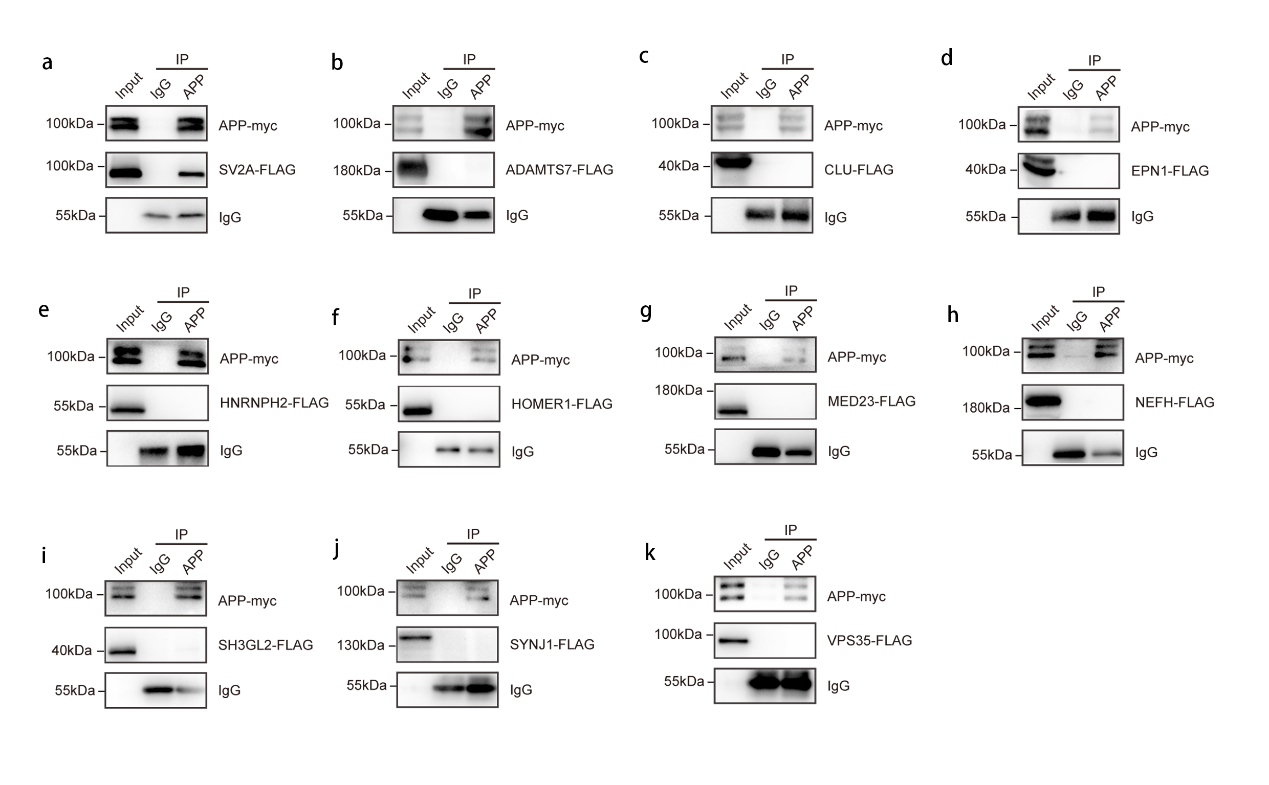
ability of APP/PS1 mice.

**Fig. S1 The Co-IP results of APP with 11 candidate binding proteins in HEK293T cells. a-k.** The Co-IP results of APP and candidate proteins in the lysates of HEK293T cells co-transfected with APP-myc/Sv2a-FLAG (**a**), App-myc/Adamts7-FLAG (**b**), App-myc/Clu-FLAG (**c**), App-myc/Epn1-FLAG (**d**), App-myc/Hnrnph2-FLAG (**e**), App-myc/Homer1-FLAG (**f**), App-myc/Med23-FLAG (**g**), App-myc/Nefh-FLAG (**h**), App-myc/Sh3gl2-FLAG (**i**), App-myc/Synj1-FLAG (**j**) or App-myc/Vps35-FLAG (**k**)
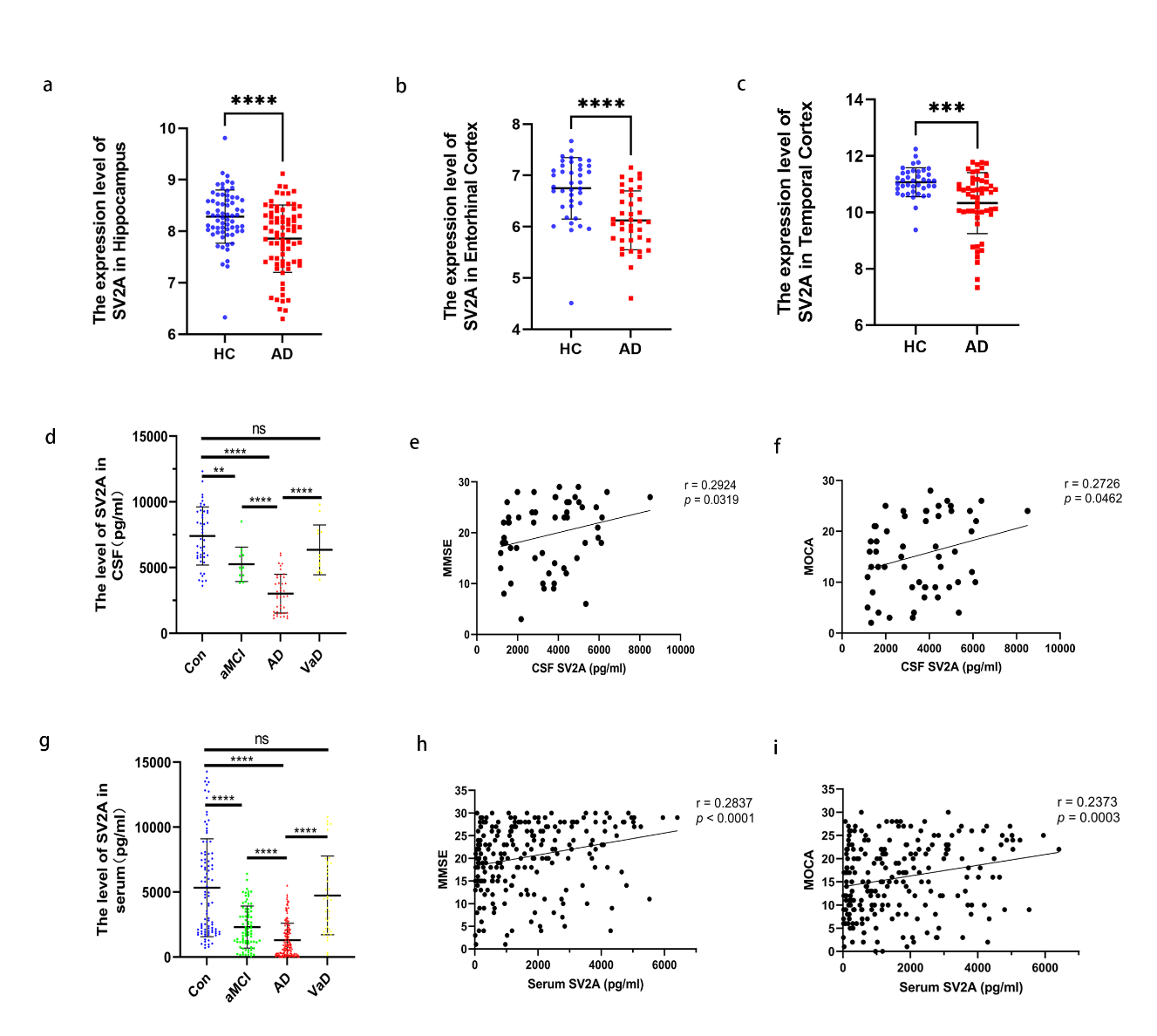
for 48-72 h.

**Fig.S2** **SV2A levels in AD patients.** SV2A expression (in TPM) in postmortem hippocampal (**a**), entorhinal cortex (**b**), and temporal cortex (**c**) samples from donors with AD and cognitively normal controls, as provided by the AlzData database. **d.** CSF SV2A levels at different stages of AD and VaD (Con = 45, aMCI = 13, AD = 41, VaD = 13). **e.** Correlation of the CSF SV2A levels with MMSE scores. **f.** Correlation of the CSF SV2A levels with MOCA scores. **g.** Serum SV2A levels at different stages of AD and VaD (Con = 95, aMCI = 80, AD = 145, VaD = 38). **h.** Correlation of the serum SV2A level with MMSE scores. **i.** Correlation of the serum SV2A level with MOCA scores. Data are presented as the means ±SD. The significance of the between-group differences was determined using the Mann-Whitney U-test. One-way ANOVA with Bonferroni post-hoc correction was applied to compare the statistical differences between multiple groups. Partial correlation analyses were performed to assess the correlations between biomarkers and cognitive scores by controlling for confounders
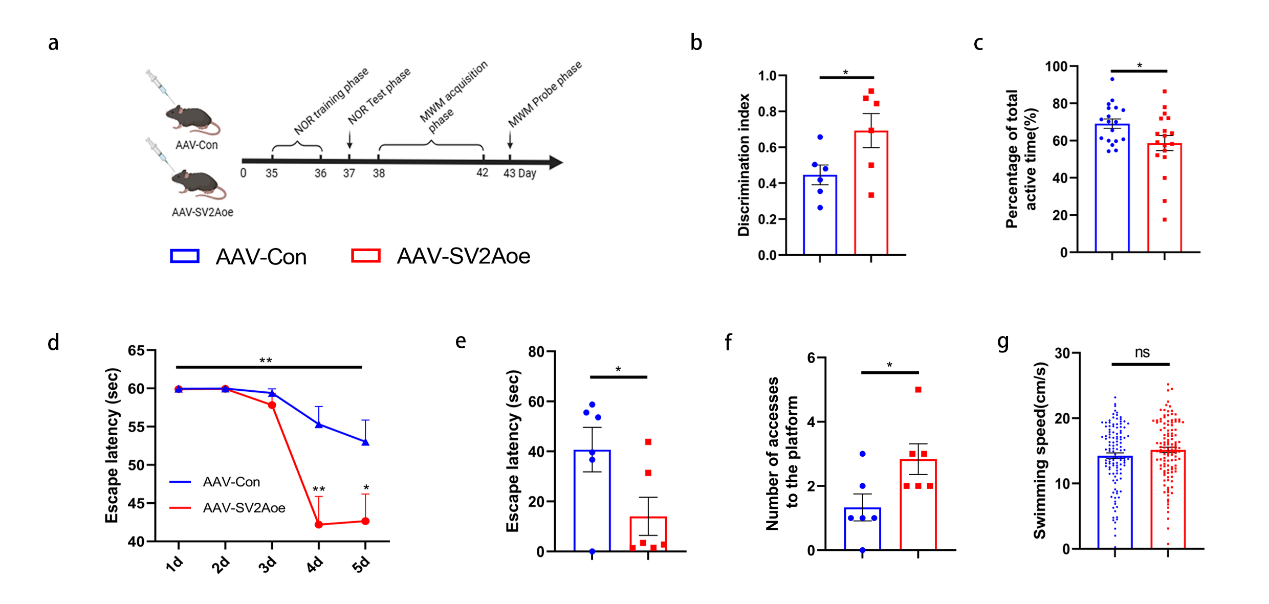
such as age and sex. **p* < 0.05, ***p* < 0.01, ****p* < 0.001, *****p* < 0.0001.

**Fig.S3 Cognitive performance in SV2A-upregulated APP/PS1 mice. a.** Timeline of behavioral experiments performed in APP/PS1 mice injected with AAV-SV2Aoe or AAV-Con. **b.** Discrimination index of the AAV-SV2Aoe injected APP/PS1 mice during the test phase NOR. **c.** Percentage of total active time of the AAV-SV2Aoe injected APP/PS1 mice during the training phase of NOR. **d.** Escape latency of the AAV-SV2Aoe injected APP/PS1 mice during the acquisition phase of MWM. **e.** Escape latency of the AAV-SV2Aoe injected APP/PS1 mice entering the original platform region for the first time during the probe phase of MWM. **f.** Number of accesses to the platform of the AAV-SV2Aoe injected APP/PS1 mice during the probe phase of MWM. **g.** Swimming speed of the AAV-SV2Aoe injected APP/PS1 mice during the acquisition phase of MWM. Data were presented as mean ± SD. All dot plots: t-test or one-way ANOVA test. * *p* < 0.05, ** *p* < 0.01, *** *p* < 0.001, **** *p* < 0.0001.

**Supplementary Table 1 Protein identification of APP-interacting by mass spectrometry analysis**

|  |  | **GST-labeled APP** | |  | **GST-labeled IgG** | |
| --- | --- | --- | --- | --- | --- | --- |
| **Gene Symbol** | **Description** | **PSMs** | **Unique Peptides** |  | **PSMs** | **Unique Peptides** |
| *Homer1* | homer protein homolog 1 isoform b | 5 | 5 |  | 3 | 3 |
| *Sh3gl2* | endophilin-A1 isoform X1 | 4 | 4 |  | 1 | 1 |
| *Synj1* | synaptojanin-1 isoform b | 4 | 4 |  | 1 | 1 |
| *Clu* | Clusterin preproprotein | 4 | 4 |  | - | - |
| *Vps35* | vacuolar protein sorting-associated protein 35 | 3 | 3 |  | 1 | 1 |
| *Nefh* | neurofilament heavy polypeptide | 6 | 3 |  | 2 | 1 |
| *Hnrnph2* | heterogeneous nuclear ribonu-  cleoprotein H2 | 8 | 2 |  | 6 | - |
| *Epn1* | epsin-1 isoform X4 | 3 | 2 |  | - | - |
| *Sv2a* | synaptic vesicle glycoprotein 2A | 2 | 2 |  | - | - |
| *Med23* | mediator of RNA polymerase II transcription subunit 23 isoform 2 | 2 | 2 |  | - | - |
| *Adamts7* | A disintegrin and metalloproteinase with thrombospondin motifs 7 isoform X4 | 2 | 2 |  | - | - |

**Supplementary Table 2 Clinical and demographic features of the diagnostic cohorts for CSF and serum SV2A**

|  | **Con** | **aMCI** | **AD** | **VaD** |
| --- | --- | --- | --- | --- |
| **CSF** |  |  |  |  |
| **No.** | 45 | 13 | 41 | 13 |
| **Sex, female/male** | 22/23 | 8/5 | 27/14 | 7/6 |
| **Age, years (SD)** | 66.40 ± 10.45 | 64.31 ± 8.60 | 62.95 ± 7.20 | 68.23 ± 6.17 |
| **MMSE score, mean (SD)** | NA | 25.77 ± 2.36 | 17.63 ± 6.40 | 17.85 ± 6.95 |
| **MOCA score, mean (SD)** | NA | 24.08 ± 1.98 | 12.59 ± 6.27 | 13.31 ± 5.40 |
| **Serum** |  |  |  |  |
| **No.** | 95 | 80 | 145 | 38 |
| **Sex, female/male** | 53/42 | 46/34 | 78/67 | 16/22 |
| **Age, years (SD)** | 65.47 ± 8.05 | 66.86 ± 7.21 | 66.74 ± 8.37 | 67.97 ± 9.24 |
| **MMSE score, mean (SD)** | NA | 27.40 ± 1.90 | 16.43 ± 6.20 | 21.89 ± 4.58 |
| **MOCA score, mean (SD)** | NA | 23.39 ± 3.54 | 11.72 ± 5.85 | 16.63 ± 4.65 |

Note: The t-test and chi-square test were used to compare statistical differences between groups in terms of age and sex, respectively. Abbreviations: AD, Alzheimer’s disease; aMCI, amnestic mild cognitive impairment; Con, control subjects; MMSE, Mini-Mental State Examination; MoCA, Montreal Cognitive Assessment; PDD, Parkinson’s disease dementia; SD, standard deviation; VaD, vascular dementia

**Supplementary Table 3 Levels of CSF and serum SV2A in aMCI, AD, and VaD**

|  | **Con** | **aMCI** | **AD** | **VaD** |
| --- | --- | --- | --- | --- |
| **CSF SV2A, mean (pg/mL) (SD)** | 7405.78 ± 2182.64 | 5252.56 ± 1258.14* | 3023.78 ± 1457.92* | 6351.24 ± 1814.95^†^ |
| **Serum SV2A, mean (pg/mL) (SD)** | 5338.34 ± 3749.43 | 2309.80 ± 1605.11* | 1302.67 ± 1296.64* | 4745.85 ± 2989.51^†^ |

Note: The normality of the distribution of the variables was assessed by the Shapiro–Wilk test. Continuous variables were compared between two independent samples using the Mann–Whitney U-test. Logistic regression models were employed to compare continuous variables between different groups before and after adjusting for covariates such as age and sex. **p* < 0.01, compared with control; ^†^*p* < 0.01, compared with AD. Abbreviations: AD, Alzheimer’s disease; aMCI, amnestic mild cognitive impairment; Con, control; SV2A, synaptic vesicle glycoprotein 2A; SD, standard deviation; VaD, vascular dementia
